# Comparative Study between Exogenously Applied Plant Growth Hormones versus Metabolites of Microbial Endophytes as Plant Growth-Promoting for *Phaseolus vulgaris* L.

**DOI:** 10.3390/cells10051059

**Published:** 2021-04-29

**Authors:** Mohamed A. Ismail, Mohamed A. Amin, Ahmed M. Eid, Saad El-Din Hassan, Hany A. M. Mahgoub, Islam Lashin, Abdelrhman T. Abdelwahab, Ehab Azab, Adil A. Gobouri, Amr Elkelish, Amr Fouda

**Affiliations:** 1 Department of Botany and Microbiology, Faculty of Science, Al-Azhar University, Nasr City, Cairo 11884, Egypt; alazharbotany5617@gmail.com (M.A.I.); mamin7780@azhar.edu.eg (M.A.A.); aeidmicrobiology@azhar.edu.eg (A.M.E.); or h.mahgoub@azhar.edu.eg (H.A.M.M.); islam79@azhar.edu.eg (I.L.); omarabdot@yahoo.com (A.T.A.); 2 Department of Biology, Faculty of Science and Arts, Al Mandaq, Albaha University, Al-Baha 1988, Saudi Arabia; 3 Department of Botany Science, Faculty of Science, Northern Border University, Arar 73211, Saudi Arabia; 4 Department of Nutrition and Food Science, College of Science, Taif University, P.O. Box 11099, Taif 21944, Saudi Arabia; e.azab@tu.edu.sa; 5 Department of Chemistry, College of Science, Taif University, P.O. Box 11099, Taif 21944, Saudi Arabia; a.gobouri@tu.edu.sa; 6 Botany and Microbiology Department, Faculty of Science, Suez Canal University, Ismailia 41511, Egypt; amr.elkelish@science.suez.edu.eg or; 7 Department of Plant Physiology, Matthias Schleiden Institute of Genetics, Bioinformatics and Molecular Botany, Friedrich-Schiller-University Jena, 07743 Jena, Germany

**Keywords:** endophytes, culture filtrate, exogenously hormone, Phaseolus vulgaris, antioxidant enzymes

## Abstract

Microbial endophytes organize symbiotic relationships with the host plant, and their excretions contain diverse plant beneficial matter such as phytohormones and bioactive compounds. In the present investigation, six bacterial and four fungal strains were isolated from the common bean (*Phaseolus vulgaris* L.) root plant, identified using molecular techniques, and their growth-promoting properties were reviewed. All microbial isolates showed varying activities to produce indole-3-acetic acid (IAA) and different hydrolytic enzymes such as amylase, cellulase, protease, pectinase, and xylanase. Six bacterial endophytic isolates displayed phosphate-solubilizing capacity and ammonia production. We conducted a field experiment to evaluate the promotion activity of the metabolites of the most potent endophytic bacterial (*Bacillus thuringiensis* PB2 and *Brevibacillus agri* PB5) and fungal (*Alternaria sorghi* PF2 and, *Penicillium commune* PF3) strains in comparison to two exogenously applied hormone, IAA, and benzyl adenine (BA), on the growth and biochemical characteristics of the *P. vulgaris* L. Interestingly, our investigations showed that bacterial and fungal endophytic metabolites surpassed the exogenously applied hormones in increasing the plant biomass, photosynthetic pigments, carbohydrate and protein contents, antioxidant enzyme activity, endogenous hormones and yield traits. Our findings illustrate that the endophyte *Brevibacillus agri* (PB5) provides high potential as a stimulator for the growth and productivity of common bean plants.

## 1. Introduction

Common beans (*Phaseolus vulgaris* L.) are important legumes as a source of food and money for small farm owners worldwide [1]. Common beans are an essential source of micronutrients and protein, in addition to their caloric content. They are of interest in developing countries as an inexpensive protein source (compared to animal protein sources) and because of their long-term storage life [2]. The popularity of this crop stems from its relative ease of production, variety of uses, good source of nutrition, and deliciousness. Beans are widely adapted to moderate temperature growing environments, with an average precipitation of approximately 400 mL and a growing season of 60–120 days [3]. By the year 2050, the world’s population is expected to increase by 34% (approximately 9.1 billion people), and thus, we need approximately 70% more food production [4].

Plant hormones, or phyto-regulators, have the leading role in a plant’s life cycle [5]. Plant hormones are involved in most of development stages such as seed germination, fruit maturation, seedling, and leaf senescence [6]. Auxin is the most common class of plant hormones, the most prominent of which is indole-3-acetic acid (IAA), a key regulator of plant growth and development [7], involved in the phototropic response, fruit development, cell differentiation, division, and elongation, and has a major role in plant physiology [7]. IAA increases stomatal conductance, the photosynthetic average, plant pigmentation, and the piling up of total soluble sugars such as fructose and glucose [8]. 

Other frequently used plant hormones are cytokinin’s, which affect cell division and many other aspects of the plant development and growth processes involving shoot growth and initiation, seed germination, abscission, senescence, and apical dominance [9,10]. Benzyl adenine (BA) is one of the commonly used cytokinin’s [11], which has promotional effects that have been demonstrated by several studies. IAA is the most common endogenous hormone and has an effective role in root elongation and growth. It results from the metabolism of L-tryptophan by various microorganisms, including plant growth-promoting bacteria (PGPB) [12].

Fungi and plant-related bacteria produce many plant hormones, including IAA, as one of the mechanisms to manage plant development and growth. Microbial indole modifies the structure of roots to boost the availability of nutrients to these microbes that can produce plant hormones [13]. 

PGPB, which have many useful effects on their host plant, involve the growth activation, metabolism regulation, and modulation of phytohormone signaling that contribute to the adaptation to adverse environmental conditions [14]. Endophytes are being studied for their ability to create novel biologically active compounds, as well as their ability to preserve plant health through their biofertilizer and biocontrol properties [15].

Endophytic fungi as symbiosis are well recognized, and approximately 1.5 million different fungal species have been identified in various host plants, with one million of them being endophytic in nature [16]. Both endophytic fungi and the host plants profit from their mutualistic relationship. Fungal endophytes have been documented to produce a variety of bioactive compounds, which improve plant tolerance to various biotic stresses [7].

Moreover, endophytes exhibit a disease-suppressive capacity, which might be induced by the production of various secondary metabolites, including salicylic acid, siderophores, enzymes, proteins, elicitors and/or antibiotics [17]. Recently, these diverse metabolites can be utilized for various biotechnological applications [18,19,20]. Beneficial endophytic bioinoculants must possess plant growth-promoting properties such as the production of ammonia, phytohormones, hydrogen cyanide, and siderophore, as well as phosphate solubilization [21]. Utilizing endophytes as biofertilizers and/or biocontrol agents in the organic farming of diverse crops has become interesting due to their richness in naturally bioactive products [22].

The efficacy of microbial endophytes in promoting plant-growth and their comparison with exogenously applied hormones have not been investigated until now. Therefore, the aim of the current investigation was the isolation, identification, and characterization of bacterial and fungal endophytes from the roots of the *Phaseolus vulgaris* plant, and the exploration of their potentiality compared to two common exogenously applied hormones on the growth and biochemical properties of *P. vulgaris* plants to explore the possibility of applying these microbial isolates as biofertilizers for the improvement of the growth performance and metabolites of crops.

## 2. Materials and Methods

### 2.1. Plant Sampling and Microbial Endophytes Isolation

Unearthed roots of the common bean (*Phaseolus vulgaris* L.) plant were collected from El-Menofia governorate (30°38′33.5″ N 30°56′54.4″ E), Egypt. Plant roots were preserved in plastic pages at 4°C for 48 h before being processed. The roots are thoroughly rinsed with tap water and then sterilized distilled water. Roots were surface sterilized by immersing them in sterile distilled water for 1 min, 70% ethanol for 1 min, 2.5 percent chlorine bleach for 4 min, 70% ethanol for 30 s, and then washing them three times in sterile distilled water. To ensure that the sterilization process is working properly; the water of last washing was streaked onto nutrient (readymade, Oxoid) and Czapek Dox (CD) (readymade, Oxoid) agar media, the absence of microbial growth in the cultured media verified the sterilization process’ success [23].

The sterilized roots were cut into small segments (5 mm/segment) aseptically, and fifteen root segments were put on three nutrient agar plates (5 segments/plate). The plates were daily observed to detect bacterial growth. The bacterial isolates that appeared around the root segment are picked up and streaked on another nutrient plate for complete purification. The same previous procedure was repeated by putting root segment on Czapek Dox (CD) agar media for fungal isolation. The fungal colony that appears around the root segment are picked up and undergo purification steps on CD agar media [24]. The purified fungal and bacterial isolates were stored on slants at 4 °C for future studies.

### 2.2. Molecular Identification of Microbial Endophytes

The endophytic bacterial and fungal isolates obtained from the surface-sterilized roots were subjected to molecular identification based on amplification and sequencing of bacterial 16S rRNA and fungal ribosomal DNA internal transcribed spacer (ITS) region. 

For bacterial identification, the genomic DNA was extracted according to the modified method [25], and the PCR protocol was achieved according to Fouda et al. [26]. Briefly, individual bacterial colonies were picked up using a sterile toothpick and resuspended in 50 µL of sterile deionized H_2_O. The cell suspension was heated for 10 min in a water bath at 97 °C, then the cell lysate was forced (15,000× *g*, 10 min) and the upper layer containing the DNA was retrieved. The content of extracted DNA was calculated by detecting its absorbance at 260 nm using a UV spectrophotometer. A fragment of 16S rDNA was PCR-amplified using the bacterial universal primers 27f (50-AGAGTTTGATCCTGGCTCAG-30) and 1492r (50-GGTTACCTTGTTACGACTT-30). The PCR tube contained 1 × PCR buffer, 0.5 mM MgCl_2_, 2.5 U Taq DNA polymerase (QIAGEN Inc., Germantown, MD, USA), 0.25 mM dNTP (Deoxynucleoside triphosphate), 0.5 µM primers, and approximately 5 ng of bacterial genomic DNA.

In the case of fungi, genomic DNA was extracted using the Gene Jet Plant genomic DNA purification Kit (Thermo procedure). Using genomic DNA as a template and primers for ITS1 (5′-TCCGTAGGTGAACCTGCGG-3′) and ITS4 (5′-TCCTCCGCTTATTGATATGC-3′), the ITS region was amplified in PCR reaction. Maxima Hot Start PCR Master Mix (Thermo), 0.5 µM of each primer, and 1 µL of extracted fungal gDNA were included in the PCR mixture (50 µL) [27].

The PCR was carried out in a DNA Engine Thermal Cycler by Sigma Scientific Services Company (Cairo, Egypt) with a hot starting performed at 94 °C for 3 min, followed by 30 cycles of 94 °C for 30 s, 55 °C for 30 s, and 72 °C for 60 s, followed by a final extension performed at 72 °C for 10 min. GATC Company (Germany) used an ABI 3730x1 DNA sequencer to perform the sequencing. Using the NCBI BLAST software, the obtained sequences were compared to the Gene Bank database. BLASTN was used to compare sequences to 16S rRNA and ITS sequences in the Gene Bank database, and bootstrap analysis was used to build phylogenetic trees. The sequences obtained in this analysis were deposited in Gene Bank under accession numbers MW485939 to MW485944 for bacteria and MW485935 to MW485938 for fungi.

### 2.3. Screening of Plant Growth Promotion Potential of Microbial Endophytes

#### 2.3.1. Colorimetric Determination of Indole-3-Acetic Acid (IAA)

Bacterial endophytes were inoculated in 20 mL of LB broth (contain g L^−1^: tryptone, 10; yeast extract, 5; NaCl, 10; 1.0 L dis. H_2_O, adjusted to pH 7) amended with 5 g L^−1^ of L-tryptophan (chosen as the best concentration from our previous study) and incubated in orbital shaker (180 rpm) at 30 ± 2 °C for 7 days. Also, fungal discs (10 mm in diameter) covered with hyphae were cultured in 20 mL CD broth containing 5 g L^−1^ of L-tryptophan and incubated in the same manner at 28 °C for 10 days. At the end of incubation period, five mL of each endophytic culture were collected and centrifuged at 6000 rpm at 4 °C for 30 min. Two mL of Salkowski’s reagent was added to 1.0 mL of each culture supernatant and measured at 530 nm (Jenway 6305 UV spectrophotometer). IAA was quantified with pure IAA standard [28].

#### 2.3.2. Ammonia Production

The potency of isolated microbial endophytes to produce ammonia in peptone broth (containing g L^−1^: peptone, 10; NaCl, 5; 1.0 L dis. H_2_O) was evaluated and detected by Nessler’s reagent. The degree of color change indicates the potency of the endophytic isolates to produce ammonia.

#### 2.3.3. Phosphate Solubilization

To determine the phosphate solubilizing strains, bacterial and fungal endophytes were cultured on Pikovskaya’s agar medium (containing g L^−1^: dextrose, 10; yeast extract, 0.5; MgSO_4_·7H_2_O, 0.1; Ca_3_(PO_4_)_2_, 5; (NH_4_)_2_SO_4_, 0.5; FeSO_4_·7H_2_O, 0.0001; MnSO_4_·H_2_O, 0.0001; KCl, 0.2; and agar 15 g/1000 mL dis. H_2_O). The development of clear zones around bacterial colonies and fungal discs considered as positive [29].

#### 2.3.4. Extracellular Enzymatic Activities

Extracellular enzymes produced by bacterial endophytes were assessed based on minimal salt (MS) media (Containing g L^−1^: KH_2_PO_4_, 1.5; NaNO_3_, 6; KCl, 5; MgSO_4_·7H_2_O, 0.5; FeSO_4_, 0.01; ZnSO_4_, 0.01; and agar 15 g/1000 mL dis. H_2_O) containing the specific dissolved indicative substrate. The inhibition zones induced around microbial endophytes growth after adding the specific reagent was measured as indicative for the enzymatic activity. Estimation of the endophytic fungal enzymes was achieved under the same conditions with addition of yeast extract (5 g L^−1^) to the MS media [30]. Endophytic enzyme activities of pectinase, amylase, xylanase, cellulase and protease were estimated by culturing the bacterial and fungal isolates on MS media containing 1% of pectin, soluble starch, xylan, carboxy-methylcellulose (CMC) and gelatin, respectively. The specific reagents hexadecyl-trimethyl ammonium bromide, iodine, absolute ethyl alcohol, and acidic mercuric chloride were used to visualize pectinolytic, amylolytic, and cellulolytic, xylanolytic, and proteolytic enzymatic activities, respectively [31].

#### 2.3.5. In Vitro Evaluation of Antagonistic Activity of Endophytic Isolates against Selected Phytopathogenic Fungi

All the endophytic isolates were screened for their antagonistic properties against the fungal phytopathogens based on dual-culture assay method [32]. The widely prevalent fungal pathogens *Fusarium* *oxysporum*, *Verticillium dahlia*, *Alternaria* *alternata* and *Pythium* *ultimum* were obtained from the Plant Pathology Department, Faculty of Agriculture, Zagazig University, Egypt. Each bacterial isolate was spotted at 3 equidistant points along the perimeter of the potato dextrose agar (PDA, readymade, Oxoid) plates and incubated in dark for 48 h at 28 °C. The fungal pathogens were previously grown on PDA at 28 °C for 7 days. Every fungal pathogen had one plug (6 mm) cut from the leading edge and inserted in the center of the incubated bacterial plates; plates without bacterial inoculation served as controls. The dual culture plates were incubated for 5 days at 28 °C, after incubation. The percentage of mycelial growth inhibition was calculated by the formula:

(1) $$\mathrm{Inhibition}\;\mathrm{percentages}\;(I\%)=\frac{R1\;-\;R2}{R1}\;\times \;100$$

where R1 is the radial growth of the phytopathogen without bacterial endophyte (control), while in endophytic inoculated plates, R2 represents pathogen radial development (dual culture plates).

Likewise, the four days-old fungal plugs (6 mm) of the endophytic isolates were inoculated in the dual culture PDA plates as the bacterial isolates.

### 2.4. Field Experiment Using Phaseolus vulgaris L. Plant

#### 2.4.1. Experimental Design

A field experiment was conducted under field conditions during the winter season of 2019 at the garden of the Faculty of Science, Al-Azhar University, Cairo, Egypt, to evaluate the impact of foliar spray of two exogenously plant hormone [i.e. indole-3-acetic acid (IAA) and benzyl adenine (BA)] compared to the metabolites of most potent endophytic bacterial strains (PB2 and PB5, separately or in a consortium (BM)) and fungal strains (PF2 and PF3 separately or in a consortium (FM)) on growth, biochemical and yield parameters of bean (*Phaseolus vulgaris* L.) plant.

The *Phaseolus vulgaris* seeds were surface disinfested by immersing them in ethyl alcohol (70%) for one minute, then in 4% sodium hypochlorite for 15 min, followed by sterilized distilled water washing.

The soil of the experimental site was classified as sandy soil containing sand, silt, and clay of 95.16%, 3.45%, 1.39%, respectively, in the 0–30 cm soil layer. The chemical compositions of soil used were Na, Ca, K, P, and Cl with values of 185.25, 25.0, 16.20, 24.30, and 132.50 mg kg^−1^, respectively. The plant was grown under field conditions and irrigated when needed. The field trial was set up in a completely randomized design (CRD). A randomized complete block design with 5 blocks was set up. Within each block were 9 plots: treated with C (control: distilled water), H1 (IAA, 100 ppm), H2 (BA, 100 ppm), endophytic metabolites (100 ppm) of PB2, PB5, BM, PF2, PF3, and FM. Each planted plot contained one row with 5 plants per treatment and 30 cm between each plant, in total 225 were grown with 45 plants for each block. For analysis, five plant samples were randomly collected per treatment.

#### 2.4.2. Culture Conditions and Extraction of Endophytic Secondary Metabolites

Endophytic bacterial isolates (PB2, PB5, and BM) were grown in LB medium for 24 h, at the end of incubation period, adjusted the bacterial concentrations at 10^8^ cell/mL. About 200 µL of the previous bacterial concentration were inoculated into 500 mL of LB broth amended with 5 g L^‒1^ tryptophan. In a shaking incubator, the cultures were incubated for 6 days at 28 °C. The bacterial broth media was centrifuged for 30 min at 6000 rpm, and the supernatant was harvested after incubation period. On the other hand, the fungal endophytes (PF2, PF3, and FM) were grown in CD agar medium at 28 °C, two fungal discs (10 mm) of a 5-day old cultures of each fungus was inoculated in 500 mL CD broth and incubated at 28 °C in shaking incubator. After incubation for ten days, fungal cultures were filtered through gauze cloth, forced for 30 min at 6000 rpm, and the supernatant was harvested.

The endophytic metabolites were extracted using the method of Kim et al. [33], with minor modifications: the obtained supernatant was mixed with an equivalent volume of ethyl acetate (1:1 *v/v*) and kept for 10 h at 4 °C, the solvent layer (containing metabolites) was separated by a separation funnel and evaporated in a rotary evaporator (40 °C/90 rpm) to get the crude metabolites. Ten mg of the concentrated residue was re-dissolved in 100 mL distilled water to get a final concentration of 100 ppm.

#### 2.4.3. Foliar Spraying of Phaseolus Vulgaris Plants with Endophytic Metabolites and Exogenously Applied Hormone

Suspensions of the exogenously hormones (IAA and BA), the endophytic metabolites (PB2, PB5, PF2, and PF3), and their consortium (BM and FM) were prepared and adjusted to a concentration of 100 ppm. During foliar spray, the soil was coated with aluminum foil to ensure that the solution only reached the tops of the plants, not the ground. Spraying was done up to down, a spray atomizer was used for foliar application, the final volume of liquid to spray the leaves was 2 mL per plant. The first spraying was done when the seedlings were 15 days old, while the second and third foliar spraying was performed when the seedlings were 30 and 50 days old, respectively. The harvest was achieved for three times described as follows: after 35 days of planting (first harvest) as a vegetative stage; after 55 days (second harvest) as a flowering stage; and the third harvest was achieved after 90 days of planting (yield stage).

#### 2.4.4. Growth and Vegetative Parameters Measurement

The shoots of 35 and 55-day-old bean plants (*n* = 5) were separated from the plants and were putted in an oven at 70 °C until a constant weight, then, the heights and dry weights of the shoots were measured. Fresh apical of shoot subsamples were stored in refrigerator for assessment the plant enzymatic activities.

#### 2.4.5. Physiological Parameters Measurements

##### Determination of Photosynthetic Pigments

The quantitative determination of chlorophylls was done using the method of Vernon and Seely [34]. One gram aliquot of fresh leaves was cut into small pieces. The pigments were extracted by grinding the cut tissue with suitable amount of glass powder in mortar using 100 mL of 80% aqueous acetone (*v/v*). The homogenate was transferred quantitatively to a Buchner filter with Whatman No. 1 filter paper. The filtrate was transferred quantitatively to 100 mL volumetric flask and made up to a total volume of 100 mL using 80% acetone.

The optical density of the plant extract was measured using spectrophotometer of two wavelengths (649 and 665 nm). These are positions in the spectrum where maximum absorption by chlorophyll (a) and (b) occurs. The concentrations of chlorophyll (a), (b) and total chlorophyll in leaf plant tissue were calculated using the following equations:

Mg. chlorophyll (a)/g tissue = 11.63 (A 665) − 2.39 (A 649)(2)

Mg. chlorophyll (b)/g tissue = 20.11 (A 649) − 5.18 (A 665)(3)

Mg. chlorophyll (a + b)/g tissue = 6.45 (A 665) + 17.72 (A 649)(4)

For carotenoids, the concentration was carried out according to equation [35]:

Car. = 1000 × (A 470) − 1.82 Chl (a) − 85.02 Chl (b)/198 = mg/g fresh wt.(5)

where (A) denotes the optical density, Chl (a) denotes the chlorophyll a; Chl (b) denotes the chlorophyll b.

##### Determination of Total Soluble Carbohydrates and Proteins

The content of total soluble carbohydrates in the dry shoot were determined using the Umbriet et al. method [36]. Contents of soluble proteins in the dry shoot were estimated according to the methods of lowery et al. [37].

##### Determination of Amylase and Protease Enzymes Activity

Amylase activities were estimated using a method modified from that described by Afifi et al. [38]. In this method, the enzyme-substrate reaction mixture consisted of 1.0 mL of the supernatant to be assayed for amylase activity and 1.0 mL of soluble starch solution (10 mg mL^−1^) into 0.5 M sodium acetate buffer pH 5.3, containing 20 mM CaCl_2_. The reaction mixture was allowed to react for 15 min at 40 °C. The reaction was stopped by adding 5.0 mL of 0.5 M acetic acid. To 1.0 mL of the incubated mixture, 3.0 mL of iodine reagent (0.01% Potassium iodide and 0.001% iodine) were added to obtain starch iodine complex, which showed blue color. The amylase activity was estimated by measuring O.D. at 660 nm. Proteases were determined using the method of Ong & Gaucher [39]. In this method, 0.25 mL of the supernatant to be assayed for proteases activities was added to 1.75 mL of 0.25% casein in 0.2 M phosphate buffer pH 7 and incubated at 37 °C for 60 min. At the end of the incubation period, 3.0 mL of 5% trichloroacetic acid was then added, and after standing at room temperature for 30 min, the solution was filtered. To 1.0 mL of the filtrate, 5.0 mL of 0.4 N NaOH and 1.0 mL diluted (1:2 *v/v*) BDH were added consecutively and after color development for 30 min. The O.D. at 660 nm was determined using UV-visible spectrophotometer.

##### Determination of Antioxidant Enzymes Activity

In 50 mL sodium phosphate buffer (pH 7.0) containing 1% soluble polyvinyl pyrrolidine (PVP), five grams of fresh leaf tissue including the terminus buds in addition to the first and second young leaves were macerated. The homogeneous agent has been centrifugated at a rate of 15,000 rpm for 20 min at 4 °C and used for enzyme activity testing. The activity of catalase (EC 1.11.1.6) enzyme was determined using the method defined by Chen et al. [40]. The mixture of final reaction (10 mL) containing 40 µL enzyme extract was added to 9.96 mL H_2_O_2_ phosphate buffer pH 7.0 (0.16 mL of 30% H_2_O_2_ to 100 mL of 50 mM phosphate buffer). The rate of change in H_2_O_2_ absorbance over 60 s was calculated using a spectrophotometer set to 250 nm and expressed in EU mg^-1^ protein. The amount of enzyme that reduced 50% of H_2_O_2_ in 60 s at 25 °C was described as one unit of enzyme activity. Peroxidase (POD, EC 1.11.1.7) activity was calculated using the method defined by Kar and Mishra [41]. In a final volume of 5 mL, the sample mixture included 125 µM phosphate buffer (pH 6.8), 50 µM pyrogallol, 50 µM H_2_O_2_, and 1.0 mL of the 20 diluted enzyme extract. The enzyme activity was expressed as EU mg^-1^ protein, and the volume of purpurogallin produced was calculated by calculating the absorbance at 420 nm.

##### Extraction, Purification, and Determination of Endogenous Hormones

The methods for extraction, purification, and determination of the hormones were carried out according to [42] with some modifications.

Hormone extraction: Around 1.0 g apical buds have been melted with liquid nitrogen; 10.0 mL acetonitrile extraction medium has been applied with an antioxidant of 50 mg L^−1^ diethyldithiocarbamate. The homogenous product was incubated at 4 °C for 12 h in the dark.The extracts were forced for 10 min at 10,000× *g*. The residue was removed with the same solvent twice.

Hormone purification: The supernatant was mixed and condensed to residual through rotatory evaporation under low pressure at 37 °C, then re-dissolved in 10.0 mL 0.5 mol L^−1^ phosphate buffer (pH 8.0), and then applied 8.0 mL chloroform and oscillated to eliminate pigment. The chloroform phase was eliminated. To eliminate hydroxybenzene, 0.15 g insoluble polyvinylpyrrolidone (PVP) was applied to the aqueous phase and centrifuged at 10,000× *g* for 10 min. Pipette 5.0 mL supernatant and switch pH to 3.0 with pure formic acid. The aqueous layer was removed twice with 3.0 mL ethyl acetate. By rotatory evaporation under low pressure, the ethyl acetate phase was condensed and re-dissolved in a 1 mL combined solution of acetonitrile/methanol/0.6 percent acetic acid solution (5:50:45, V:V:V). Finally, 0.22 mm hydrophobic membranes were used to filter the hormone extraction.

Hormone determination: A high-performance liquid chromatography (HPLC) method was used to analyze the hormones. For loading into a Waters symmetry C18 column (4.6 mm × 150 mm, 5 mm), samples (20 µL) were loaded into a specific 20 µL loop. Acetonitrile: methanol: 0.6% acetic acid solution (5:50:45, V:V:V) have been used as a mobile phase. A Waters series 515 pump was used to elute samples from the column at a flow rate of 0.6 mL min^−1^ at 25 °C. Hormone peaks were observed using a photodiode array detector (Waters 2998 Sparations Module, USA) with absorbance set at 218 nm for IAA, 270 nm for BA, 200 nm for GAs, and 262 nm for ABA.

Hormone quantification: By comparing the retention time and characteristic absorption wavelength of various standard hormones (Sigma Chemical, Co., St. Louis, MO, USA), the sample hormones were identified. The hormone concentrations were determined by comparing the peak areas of the samples to those of standard samples. Both of the results were adjusted to account for the known recovery rates of various hormone concentrations. BA, IAA, GAs, and ABA had recovery rates of 86.35 ± 3.17, 84.79 ± 3.48, 83.65 ± 4.15, and 88.01 ± 2.98%, respectively (calculating process of the recovery rate: two equivalent samples were taken, one of which was added with a quantitative reference material of the component to be checked, and then the two samples were purified and measured simultaneously using the same procedure). The recovery rate was calculated as the ratio of the difference between the two samples’ measured quantities and the added standard material quantity.

##### Yield Parameters Measurements

The number and weight of pods per plant were measured after 90 days of sowing from five single plants of the row in each plot. The number and weight of seeds per pod were assessed from five single plants of the row in each plot. One hundred-seed weight (g) was determined using a sensitive digital balance. Contents of the total soluble carbohydrate and the total soluble protein were determined in seed yield that are calculated form the five single plants of the row of each plot using sensitive digital balance and corrected based on seed moisture content.

### 2.5. Statistical Analysis

Data were statistically analyzed using SPSS v17 (SPSS Inc., Chicago, IL, USA). One-way analysis of variance (ANOVA) was used to evaluate the In-vitro antagonistic activity, extracellular enzymes production, IAA, and ammonia production potential, P-solubilization ability of endophytic isolates and comparing the effect of spraying the metabolites of these endophytes and spraying exogenously IAA, BA on *Phaseolus vulgaris* growth performance. Tukey’s range tests were used to do an a posteriori multiple comparison at *p* < 0.05. As mentioned above, all analyses are the averages of three to six independent replicates.

## 3. Results and Discussion

### 3.1. Isolation and Identification of Bacterial and Fungal Endophytes Isolated from Root of Common Bean Plant

**Figure 1:**
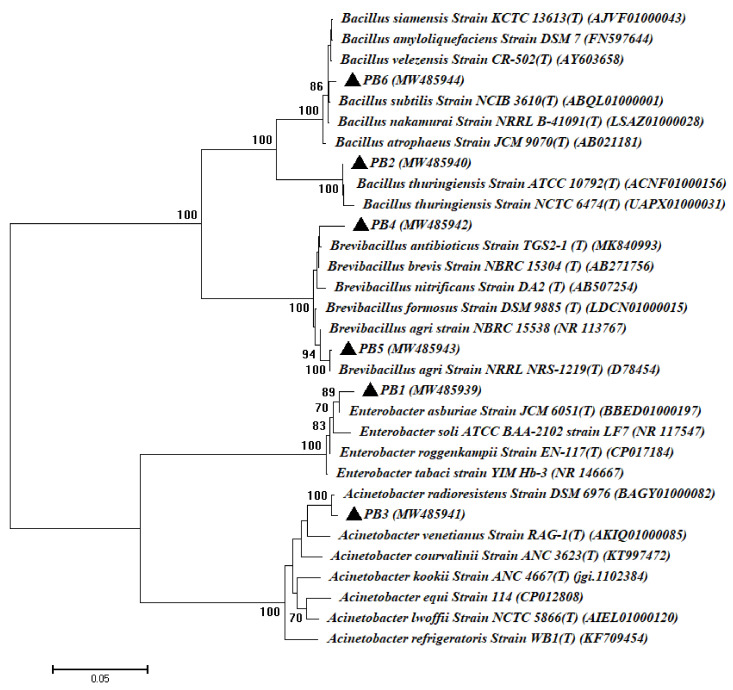
Phylogenetic tree of the 16S rRNA sequences of bacteria isolated from the root of the common bean plant. PB1–PB6 refers to the 16S rRNA sequences of bacterial isolates. The bacterial isolates and their identities were listed in Table 1. MEGA 6 was used to carry out the analysis, which used the neighbor-joining approach with a bootstrap value (1000 replicates).

In this study, a total of twenty-five bacterial isolates and twelve fungal isolates were isolated from the surface-sterilized roots of common bean (*Phaseolus vulgaris* L.) as culturable endophytes (as mentioned in the material and method section). Out of bacterial and fungal isolates, six bacterial species (PB1, PB2, PB3, PB4, PB5, and PB6) and four fungal species (PF1, PF2, PF3, and PF4) were selected as different strains based on morphological and cultural characteristics, as well as microscopic examination. These isolates were subjected to molecular identifications based on gene sequence analysis of 16S rRNA for bacterial isolates and internal transcribed spacers (ITS) for fungal isolates. The gene sequence analysis revealed that the isolated bacterial endophytes were related to two phyla. The Proteobacteria (class: γ-Proteobacteria) and Firmicutes (class: Bacilli) had, percentages of 33.3% and 66.7%, respectively. The γ-Proteobacteria strains were identified as *Enterobacter asburiae* PB1 and *Acinetobacter radioresistens* PB3, while the Firmicutes strains were identified as *Bacillus thuringiensis* PB2, *Brevibacillus brevis* PB4, *Brevibacillus agri* PB5, and *Bacillus subtilis* PB6 with similarity percentages ranging between 98.88% and 99.84% (Table 1, Figure 1).

Consistent with our data, López-López et al. [43] recorded *Bacillus* spp. and *Acinetobacter radioresistens* as bacterial endophytes isolated from the surface-sterilized root of *Phaseolus vulgaris*. Recently, Sendi et al. [44] showed that most bacterial endophytes associated with the roots of the common bean belong to *Bacillus* spp. with a percentage of 58.33%. Moreover, the bacterial endophytes isolated from sterilized common bean leaves are related to the Proteobacteria (36.7%), Firmicutes (32.9%), and Actinobacteria (29.7%) of the total bacterial isolates [45]. The *Bacillus* species are considered the most predominant endophytic strains isolated from different plant species, having been isolated from *Glycyrrhiza* spp., *Lycium chinense*, *Belamcanda chinensis*, *Pinellia ternata*, *Achillea fragrantissima*, *Taxus yunnanensis*, *Bletilla striata*, *Pulicaria incisa*, *Fagonia mollis*, *Leonurus heterophyllus*, *Digitalis purpurae* and *Pinellia pedatisecta* as described previously [29,46].

**Figure 2:**
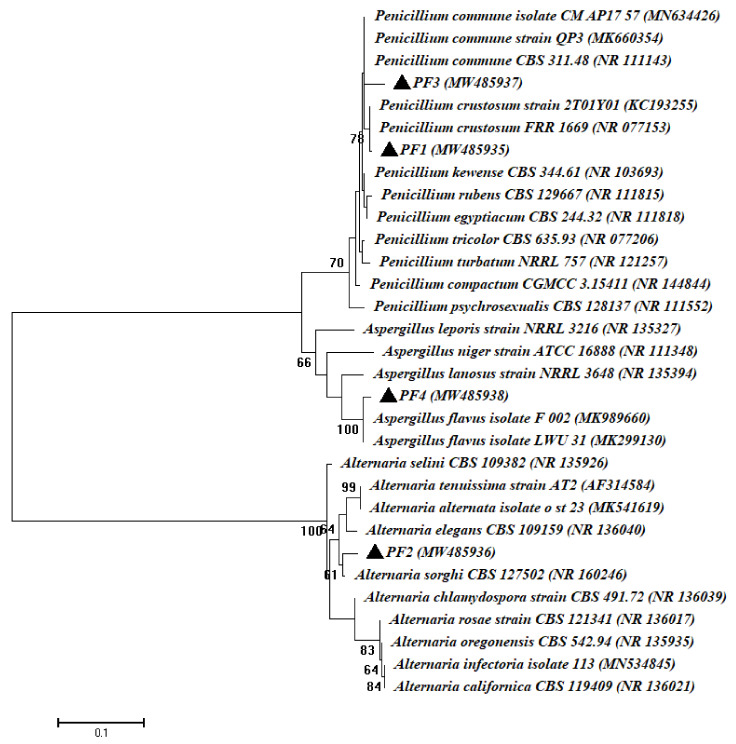
Phylogenetic tree of fungal strains’ ITS sequences against NCBI reference sequences (National Center for Biotechnology Information). PF1–PF4 refers to the ITS sequences of fungal isolated from common bean plants. The fungal isolates and their identities were listed in Table 1. The analysis was carried out in MEGA 6 using the neighbor-joining approach.

In the current study, four fungal endophytic strains were obtained from the sterilized root of the *Phaseolus vulgaris* plant belonging to Ascomycota, defined as *Penicillium crustosum* PF1, *Alternaria sorghi* PF2, *Penicillium commune* PF3 and *Aspergillus flavus* PF4, with identification percentages ranging between 98.63% and 99.62% (Table 1, Figure 2). To the best of our knowledge, this is the first record of *Alternaria sorghi* being isolated as an endophytic strain. In our recent study, *Penicillium* spp., *Alternaria* spp., and *Aspergillus* spp. were identified as endophytic fungi isolated from the medicinal plant *Ephedra pachyclada* [47]. These fungi have a variety of plant growth-promoting properties, including IAA production, antimicrobial activity, ammonia production, and phosphate solubilization. To date, there have been few published studies concerning fungal endophytes isolated from the root of *Phaseolus vulgaris* as plant growth-promoting. 

### 3.2. In Vitro Assessment of the Plant Growth-Promoting Activities of Bacterial and Fungal Endophytes

#### 3.2.1. Colorimetric Determination of IAA

**Figure 3:**
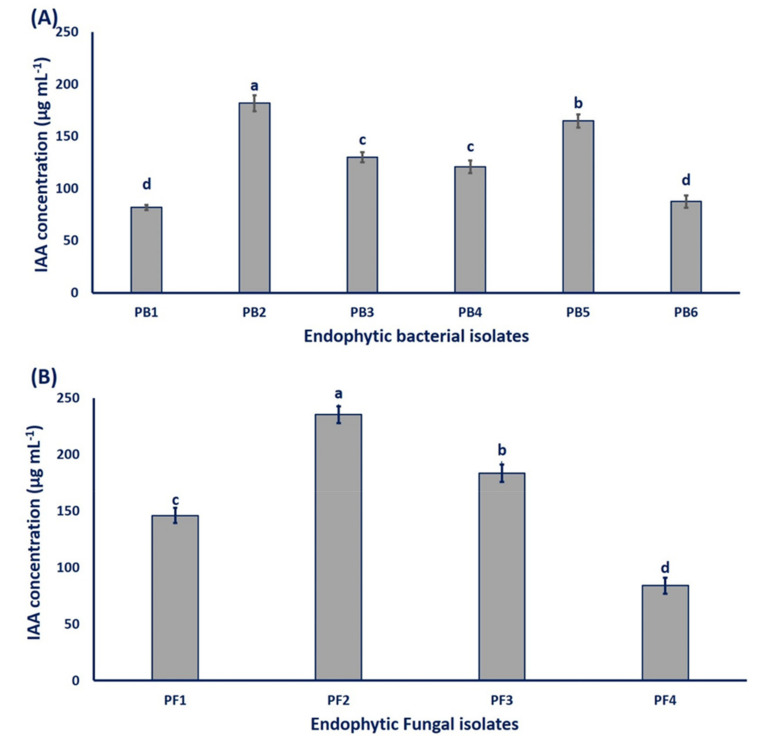
IAA production by (**A**) endophytic bacterial isolates and (**B**) endophytic fungal isolates; with 5 g L^−1^ tryptophan. Bars with the same letter for each endophytic isolate did not differ significantly at a significant level of (*p* ≤ 0.05); Error bars indicate means ± SE by LSD test (*n* = 6). PB1 is *E. asburiae;* PB2 is *B. thuringiensis*; PB3 is *A. radioresistens*; PB4 is *B. brevis*; PB5 is *B. agri*; PB6 is *B. subtilis*; PF1 is *P. crustosum*; PF2 is *A. sorghi*; PF3 is *P. commune*; PF4 is *A. flavus*.

Our results showed the ability of all the endophytic isolates to produce IAA to varying degrees in media containing tryptophan (5 g L^−1^). The fungal endophytes exhibited higher bio-fabrication of IAA than those made by endophytic bacteria. The fungal endophytes of *Alternaria sorghi* PF2 and *Penicillium commune* PF3 displayed the maximum IAA production, recording 235.33 ± 7.51 and 183.33 ± 7.53 µg mL^−1^, respectively, while elevated bacterial IAA synthesis was carried out by isolating *Bacillus thuringiensis* PB2 and *Brevibacillus agri* PB5, with values of 181.66 ± 7.62 and 164.66 ± 6.38 µg mL^−1^, respectively (*p* ≤ 0.001; Figure 3). In the same regard, ALKahtani et al. [29] reported the potency of the endophytic *Brevibacillus* sp. for production of IAA (59.7 µg mL^−1^) in media containing 5 mg mL^−1^ of tryptophan. Moreover, HPLC analysis confirmed IAA production by the endophytic fungi *Colletotrichum fructicola* associated with *Arabica coffee*, recording 1205.58 µg mL^−1^ after 26 days of incubation [48]. Previous studies have indicated the ability of microbial endophytes to produce IAA to varying degrees, whereas in the current study, isolated endophytes produced IAA in the range of 81.6 ± 2.6–235.3 ± 7.5 µg mL^−1^. IAA has a vital role in plant microbial interactions; it has been suggested as a communication method between the endophytic microbiome and the roots of the host plant [49]. Therefore, our isolates may affect plant growth and are candidates for use as surrogates to improve crop quality and productivity.

#### 3.2.2. Ammonia Production and Phosphate Solubilization

Adding nitrogen fertilizers to the soil is costly, so ammonia-producing endophytes that are likely to supply plants with nitrogen constitute a desirable feature of soil fertility and improved plant growth. Additionally, ammonia production helps plants resist colonization by pathogens [50]. Herein, a varying degree of ammonia production capacity was observed in 50% of the bacterial isolates *Bacillus thuringiensis* PB2, *Brevibacillus brevis* PB4, and *Brevibacillus agri* PB5, and in 75% of the fungal isolates *Penicillium crustosum* PF1, *Alternaria sorghi* PF2, and *Penicillium commune* PF3 (Supplementary Table S1). The high proportion of isolates producing ammonia in this study is consistent with the study conducted by Szilagyi-Zecchini et al. [51]. Most of the endophytic bacteria of the genera *Pseudomonas*, *Enterobacter,* and *Bacillus* isolated from chickpea plants have been identified as ammonia producers [52]. Recently, Ripa et al. [53] isolated and identified the endophytic fungal inhabitance of wheat plants and declared that 34% of the isolated fungi displayed ammonia production activities; these isolates belong to the genera *Aspergillus*, *Fusarium*, *Alternaria* and *Trichoderma*. Endophytic bioinoculants must possess plant growth-enhancing properties such as hydrogen cyanide, phytohormones, siderophores, ammonia production, and phosphate solubilization [21].

With the exception of nitrogen, phosphate is the most important macronutrient needed by plant, and despite the abundance of phosphates in agricultural soil, plants do not have access to them, because they are incorporated into organic compounds or in the form of mineral salts of scarce solubility [54]. Therefore, we explored the ability of our isolates to dissolve phosphate and the possibility of using them as alternatives for sustainable agriculture. The phosphate‒solubilizing activity of isolated endophytes was indicated by the clearance zones developed on Pikovskaya’s media containing tricalcium phosphate. Three bacterial isolates and three fungal isolates were potent phosphate solubilizers. However, the fungal endophytic isolates manifested higher phosphate‒solubilizing capacity than the bacterial endophytes, with the highest phosphate‒solubilizing activity listed for fungal isolates *Alternaria sorghi* PF2 and *Penicillium commune* PF3 (Supplementary Table S1).

In the same regard, 11 fungal isolates associated with wheat plants (*Triticum aestivum* L.), showed phosphate‒solubilizing potential to varying degrees and were defined as phosphate solubilizers based on the halo zones that were developed around the fungal growth on Pikovskaya’s medium [53]. Interestingly, one of the most essential properties of endophytes is their ability to dissolve insoluble phosphate in the soil. Haidar et al. [55] isolated 27 endophytes of bacteria from the endosphere of *Corchorus olitorius* and specified the isolates, *Bacillus* sp., *Bacillus firmus*, *Staphylococcus pasteuri* and *Kocuria* sp. as robust phosphate solubilizers, and they estimated the solubilized phosphate in broth media in the range of 113.52 ‒ 218.47 μg P/mL. Microorganisms dissolve inorganic phosphate by acidification due to the production of inorganic and organic acids and the excretion of H^+^, in addition to siderophore and exopolysaccharide production, while organic phosphate solubilization is mediated by phytase, C–P lyase, and phosphatase enzymes [56].

#### 3.2.3. Extracellular Enzymatic Activities

Practically, fungal and bacterial endophytes produce various key enzymatic groups such as tyrosinase, xylanases, amylases, pectinases, hemicellulases, gelatinase, phytases, cellulases, proteases, chitinase and asparaginase. The majority of these enzymes are produced by the endophytes residing in crops or medicinal plants, and the enzymatic activities of these endophytes have been detected based on agar-based procedures [57]. 

The bacterial and fungal endophytic isolates showed different activities in the production of one or more of the tested enzymes. In general, the fungal isolates produced more enzymes than the bacterial isolates; this was evidenced by recording larger clear degrading sectors than those recorded for the bacterial isolates on the indicative substrate plates. Bacterial isolates *Bacillus thuringiensis* PB2 and *Brevibacillus agri* PB5 produced the highest amounts of amylase, cellulase, and protease, while the isolate *Acinetobacter radioresistens* PB3 was the largest producer of bacterial pectinase and xylanase (*p* ≤ 0.001). The maximum fungal enzymatic production was assigned to the isolate *Bacillus thuringiensis* FB2 with degrading clear zones of 37 ± 1.7, 42 ± 1, 38.3 ± 1.2, 42.3 ± 1.3, and 31.3 ± 0.8 mm for amylase, cellulase, protease, pectinase, and xylanase respectively (Supplementary Table S1).

Compatible with our results, ALKahtani. et al. [29] isolated 13 culturable bacterial endophytes from plants that have therapeutic properties, namely, *Fagonia mollis* Delile and *Achillea fragrantissima* (Forssk) Sch. Bip. These isolates belong to genera *Bacillus*, *Paenibacillus* and *Brevibacillus* and showed varying activity of pectinase, xylanase, CMCase, gelatinase, cellulase and amylase. Furthermore, 52 endophytic fungal isolates were obtained from *Thai orchids*; at least 25% of these isolates showed enzymatic activity by the production of one or more of the exo-enzymes: Lipase, amylase, protease, cellulase and pectinase [58]. Endophytic enzymes play a major role in the hydrolysis processes that aid their colonization of the selected host plant and then gain their food from the plant tissues and their interactions with plant pathogens [59]. On the contrary, during long periods of co-evaluation, plants may try to limit the growth of endophytes by producing many toxic substances, but endophytes try to gradually form some tolerance mechanisms by producing mycotoxins and exoenzymes. In addition, these enzymes are valuable in many industrial, agricultural, and medical applications [60].

#### 3.2.4. In Vitro Antagonistic Activities

Previous studies have given increasing attention to the endophytic microbes as a distinct choice for the biological control of some plant diseases for being environmentally safe. Likewise, our results showed the potency of all the isolated endophytes to reduce the radial growth of the phytopathogenic fungi cultured on dual culture plates. The bacterial and fungal isolates inhibited more than 20% of the fungal growth of *Fusarium oxysporum*, *Alternaria alternata, Verticillium dahlia*, while *Pythium ultimum* growth was reduced by more than 9%. The maximum growth inhibition of *A. alternata* and *V. dahlia* was assigned to the bacterial isolate *Brevibacillus agri* PB5, recording antagonistic indices of 51.6 ± 0.5% and 62.6 ± 2%, respectively. The fungal isolate *Penicillium commune* PF3 showed the highest antagonistic activity against *F. oxysporum* with an inhibition percentage of 61.3 ± 2.3%. In addition, the extreme suppression of *P. ultimum* (55.3 ± 2.6%) was attributed to the fungal isolate *Alternaria sorghi* PF2 (Table 2).

In agreement with our findings, Egamberdieva et al. [61] isolated endophytic bacteria from plants with therapeutic properties, that is, *Hypericum perforatum* and *Ziziphora capitata*, the isolates of which belonged to the genera *Stenotrophomonas*, *Pantoea*, *Achromobacter*, *Serratia*, *Enterobacter*, *Bacillus*, *Erwinia*, *Arthrobacter,* and *Pseudomonas* and showed diverse antagonistic activity against a group of pathogenic soil-borne fungi, including the genera *Fusarium*, *Botrytis*, *Pythium*, *Gaeumannomyces* and *Alternaria*.

Bacterial endophytes should have various properties that manage the control of phytopathogens such as siderophores, antibiotics and antifungal production, the secretion of extracellular enzymes that degrade fungal cell wall and fusaric acid, and the synthesis of volatile organic components, in addition to the induction of host plant‒induced systemic resistance [62].

In addition, various investigations have reported the contrasted antagonistic activity of different fungal endophytes against the phytopathogenic fungi; *Fusarium solani*, *Ganoderma boninense*, *Sclerotinia sclerotiorum* and *Colletotrichum acutatum* [63,64].

### 3.3. Evaluation of the Promotion Effect of the Most Potent Bacterial and Fungal Endophytic Strains on the Growth and Metabolic Activities of the Phaseolus vulgaris Plant under Field Conditions

A field study was performed in the Botany and Microbiology Department, Faculty of Science, Al-Azhar University, Cairo, Egypt, during the winter season in 2019 to investigate the effect of two exogenously plant growth-promoting hormones (IAA and BA) compared to the metabolites of the most potent bacterial (*Bacillus thuringiensis* PB2 and *Brevibacillus agri* PB5) and fungal (*Alternaria sorghi* PF2, *Penicillium commune* PF3) endophytic strains isolated from the roots of common bean plants, or their combination (BM and FM) as a foliar spraying on the growth and metabolic activities of *Phaseolus vulgaris* L. plants at three harvested stages. The first harvest (35 days of planting) as a vegetative stage; the second harvest (55 days) as a flowering stage; the third harvest as a yield stage (90 days of planting). The vegetative growth parameters, as well as all physiological parameters, were analyzed from the first and second harvest, whilst the yield parameters were analyzed only from the third harvest.

The foliar spraying approach is preferring over other treatment method such as soaking because of symmetrical spread of different treatments over the whole crop, in addition, the spraying method increase the response of plant to treatments. Recently, the spraying method was more effective than the soaking method during the study of CuO-NPs on the morphological and physiological growth traits of *Triticum aestivum* L. [65]. Moreover, Sarkar et al. [66] showed that the foliar spraying of micronutrients was more effective due to it is overcomes the losses of nutrient in soil amendment other treatment methods.

#### 3.3.1. Effect of the Foliar Spraying of Exogenously Applied Hormone and Microbial Endophyte Metabolites on the Vegetative Growth Traits of Common Bean Plants

Foliar spraying of common bean plants with 100 ppm of IAA or BA led to a non-significant increase in shoot and root lengths compared to control plants. However, plants treated with IAA showed a non-significant decrease in root length at the first stage, while the shoots of plants treated with BA significantly increased in the first stage of growth compared to non-treated plants. On the contrary, plants treated with the metabolites of the endophytic isolates displayed a significant increase in shoot length during the first stage of growth, and the root length significantly increased in the second stage of growth compared to control plants. Neither exogenously hormones nor bacterial metabolites had a significant effect on the leaf number of plants with respect to control plants (Table 3). In the same context, Turbat and colleagues performed a plant bioassay to investigate the effect of fungal endophytic extracts on some of the growth parameters of *Arabidopsis thaliana* and recorded a boost in the length of the primary root, and the significant modifications in the photosynthetic pigments were due to endophytic fungal extract treatments. However, they reported a decrease in the plant biomass [67]. Moreover, the fermentation filtrate from the endophytic bacterial complex (*Pestalotiopsis* aff. *neglecta*/*Luteibacter* sp.) significantly increased the length of tomato seedlings with considerably longer roots compared to controls in a seedling assay conducted in vitro in petri dishes [68].

Furthermore, foliar spraying with exogenously applied hormones or endophytic extracts resulted in a comparable increase in the biomass (fresh and dry root and shoot weight) of treated plants at all stages of development, and this increase was significant in most treatments. However, the root fresh weight of the *Penicillium commune* (PF3) and fungal consortium (FM) treatments recorded insignificant weight loss, while the root dry weight of *Alternaria sorghi* (PF2) plants significantly decreased in the first stage of growth compared to controls (Supplementary Table S2). 

Analysis of the pure culture filtrates of the fungal endophytes *Phoma glomerata* LWL2 and *Penicillium* sp. LWL3 proved the existence of various concentrations of gibberellins (GAs) and IAA. Moreover, the application of these culture filtrates significantly improved the shoot length, chlorophyll content, and fresh and dry weight of shoots of both the dwarf mutant Waito-*C* (GAs-deficient) and the common rice cultivar (Dongjin-beyo) [69]. The endophytic fungi associated with mangroves were also evaluated for their ability to promote *Oryza sativa* L. growth. All of the isolated endophytic fungi were found to promote the growth of *O. sativa* L. “Cempo Ireng” [70]. Concerning the effect of exogenously applied hormones on plant growth, spraying exogenous auxins and cytokinin such as IAA and 6-benzylaminopurine resulted in an asymmetrical response of the treated alfalfa plants during their vegetative growth, while their application as a mixture was most effective and increased the biomass of plant shoots; however, the clearest effect was for the rate of total chlorophyll to carotenoid content [71]. Some endophytic fungi isolated from maize and rice plants, such as *Fusarium*, *Sarocladium*, *Aspergillus* and *Penicillium*, have been confirmed to be determining factors in plant growth improvement [72]. Fouda et al. [31] found that *Penicillium chrysogenum* and *Alternaria alternata* endophytic fungi, isolated from the *Asclepias sinaica* plant, experience enhanced root growth and root elongation attributed to the production of ammonia and IAA. Abdallah et al. [73] investigated the ability of endophytic bacteria isolated from *Withania somnifera* fruits to promote plant growth. According to the findings, the most active isolate, *Alcaligenes faecalis*, was found to produce indole-3-acetic acid and improve phosphate solubilization.

Generally, microbial endophytes can be used as biofertilizers, which reduce the reliance on chemical fertilizers while also increasing nutrient availability, thus enhancing plant growth.

#### 3.3.2. Effect of the Foliar Spraying of Exogenously Applied Hormones and Microbial Endophyte Metabolites on the Chlorophyll and Carotene Contents of Common Bean Plants

The photosynthetic is a key process for life, it is meaning consumption of solar energy and transformed to energy for chemical compounds synthesis, also, it is strongly affected by various environmental stressors such as drought, high or low temperature, salinity, and intensity of light [74]. Plant photosynthetic potential is correlated with chlorophyll concentration, which provides some insight into the physiological state of the plant system. Regarding chlorophyll a (Chl. a), no treatments had a significant effect except for BA treatment, which led to a significant increase in the Chl. a content during the two harvest stages. In agreement with our results, Hanaa and Safaa [75] concluded that foliar application of IAA (100 ppm) can enhance the chlorophyll content of *Triticum aestivum* L. (bread wheat). Ma and co-authors used chlorophyll and carotenoids as indicator for investigate the effect of exogenous application of abscisic acid and 1-naphthaleneacetic acid on the coloration of fruits [76].

In addition, the metabolites of *P. commune* (PF3) were the only treatment that significantly increased chlorophyll b (Chl. b) in the first harvest stage, whereas in the second harvest stage, plants treated with IAA, PB5, BM, and PF3 showed a significant increase in their Chl. b content. The total chlorophyll content was increased for plants treated with *B. thuringiensis* (PB2) and *P. commune* (PF3) in the first harvest stage, while the following treatments of PB5, BM, PF3, and FM positively affected the total chlorophyll content of plants in the second harvest stage. These findings are consistent with those of Heidari and Golpayegani [77], who discovered that rhizobacteria (*Pseudomonades* sp., *Bacillus lentus*, *Azospirillum brasilens* and their combinations) improved photosynthetic pigments in basil (*Ocimum basilicum* L.) plants, where a combined inoculation of *Pseudomonas* sp. and *Bacillus lentus* in plant stimulated chlorophyll synthesis as well as photosynthetic electron transport. Timmusk et al. [78] found that inoculating wheat seedlings with *Bacillus thuringiensis* AZP2 under drought stress resulted in increased plant biomass and five-fold higher survival under extreme drought due to lower volatile emissions and increased photosynthesis, implying that bacterial inoculation enhanced plant stress resistance. Naveed et al. [79] reported that *Burkholderia phytofirmans* strain PsJN and *Enterobacter* sp. FD17 increased plant biomass and photosynthetic rate of the Mazurka maize cultivar up to 48% and 45%, respectively, as compared with the control under normal irrigation. Kumar et al. [80] observed a decline in chlorophyll content in chickpea leaves due to drought stress; however, inoculation with PGPR mitigated the negative effects of drought on chlorophyll content.

Based on our results, none of the treatments appeared to have effect on the plant carotene contents in the first harvest stage. However, common bean plants treated by BA, PB2, PF2, PF3, and FM experienced slight increases in plant carotene in the second stage, while IAA, PB5, and BM negatively affected plant carotene content **(**Supplementary Table S3).

The growth stimulatory effect of IAA and BA could improve plant nutrient content, so the augmented pigmentation in our treated plants could be attributed to a potential increase in Mg and the other essential elements required for chlorophyll synthesis, in addition to down-regulation of the chlorophyll degradation process [81]. Moreover, the foliar spraying of IAA (10^‒8^ M) elevated the chlorophyll level of *Brassica juncea* by 13.27%, 18.90%, and 17.87% after 30, 45, and 60 days, respectively, from sowing [82]. In addition, enhanced growth involves increased leaf area and a consequent improvement in photosynthetic properties [82], keeping in mind the role of cytokinin’s in boosting the activity of the ribulose-1, 5-bisphosphate carboxylase/oxygenase (Rubisco) enzyme in the photosynthesis process, raising the photosynthetic capability [83]. Sosnowski et al. [71] observed that cytokinin increased the content of plastid pigments in alfalfa leaves. However, a combination of auxin and cytokinin increased nitrate reductase production in alfalfa roots and increased the ratio of overall chlorophyll content to carotenoids.

#### 3.3.3. Effect of the Foliar Spraying of Exogenously Applied Hormones and Microbial Endophyte Metabolites on the Total Soluble Carbohydrate and Protein Contents of Common Bean Plants

Our results indicate that all treatments, whether of exogenously applied hormones or endophytic metabolites, led to a significant increase in the carbohydrate and protein contents of the roots and shoots in all treated plants during the two growth stages. However, the plants treated with IAA, BA, or the *Bacillus thuringiensis* PB2 and *Alternaria sorghi* PF2 endophytes led to an insignificant increase in root proteins in both growth stages. Moreover, the plants sprayed with the endophytic culture filtrates manifested maximum increases in carbohydrate and protein contents in all growth stages, while plants treated with BA showed a maximum increase in the root proteins for all growth stages (Table 4). 

Frequent studies have reported the favorable effect of applying exogenously applied hormones on the contents of soluble carbohydrates and proteins in plants. In this context, treating the in vitro cultures of *Aechmea blanchetiana* with the auxins 1-naphthaleneacetic acid (NAA) and indole-3-butylic acid (IBA) enhanced the dry and fresh mass of the roots. Moreover, the roots of the 70-day-old plantlets treated with IBA showed increased concentrations of its soluble protein content. After 90 days of culture, the roots of the plantlets treated with NAA displayed high concentrations of soluble carbohydrates [84]. In general, sucrose is the main photosynthetic product; it is the essential substrate of sink metabolism and the major form of transported carbon. Thus, IAA, BA, and endophytic extracts have a fundamental role in increasing the carbohydrate content of sprayed plants [85].

Our results confirm the findings of Ismail et al. [86], who reported that the association between the fungal endophyte *Aspergillus japonicus* and the roots of soybeans causes a considerable increase in growth traits and significantly increased the plant content of total sugars, lipids, and proteins under normal and heat-stressed growth conditions. Existing evidence suggests the role of cytokinin’s and auxin in managing endosperm cell division through the grain-filling phase in rice. Thus, these leading hormones might control the sink size (grain capacity) for carbohydrate accumulation [87]. Moreover, Bhatia and Singh [88] used ^14^C and proved the favorable role of IAA in transforming sugars into starch in grain.

#### 3.3.4. Effect of the Foliar Spraying of Exogenously Applied Hormones and Microbial Endophyte Metabolites on the Amylase, Protease, Catalase, and Peroxidase Activities of Common Bean Plants

The common bean plants treated with 100 ppm IAA manifested a significant increase in catalase and peroxidase activities in both growth stages. Meanwhile, protease activity showed a non-significant increase, and although amylase activity showed a non-significant increase in the first stage of growth, it experienced a significant increase in the second stage of growth. On the contrary, *P. vulgaris* plants sprayed with 100 ppm BA showed a significant increase in peroxidase activity in all growth stages, as well as a significant decrease in catalase activity in the second growth stage.

Interestingly, the common bean plants treated with endophytic metabolites displayed varying degrees of enzymatic activities. Plants sprayed with 100 ppm PB5 showed a maximum increase in amylase, protease, catalase, and peroxidase activities in all growth stages. The obtained results in Table 5 clearly show the insignificant responses of protease activity to microbial endophytes individually or mixed at both stages of growth. In harmony with our results, Sadar et al. [89] determined that the crown leaves and fruit pulp of pineapples (*Ananasa comosus*) harbor 28 fungal endophytes belonging to the genera *Aspergillus, Penicillium, Cladosporium, Fusarium, Colletotrichum* and *Alternaria*. These fungal endophytes produce one or more of the enzymes amylase, protease, and lipase in varying degrees. Extracellular enzymes are the byproducts of microbial cell formation, and they play a role in a variety of biological and environmental processes outside of cells. Plants use antioxidant enzymes such as catalase and peroxidase to ameliorate the toxic substances such as reactive oxygen species (ROS), biotic, and abiotic stresses [90].

In reality, the xylanase, hemicellulase, phytase, protease, asparaginase, cellulase, pectinase, tyrosinase, gelatinase, chitinase, amylase, and other enzymes formed by the endophytic bacteria and fungi that inhabit medicinal or crop plants, are among the most important enzymes [57].

#### 3.3.5. Effect of the Foliar Spraying of Exogenously Applied Hormones and Microbial Endophyte Metabolites on the Endogenous Hormones of Common Bean Plants

Exogenous hormones play an important role in stimulating plant growth under various biotic and abiotic stresses, but little is known about the relationship of exogenous hormones to their endogenous concentrations [91]. Our tests showed that the application of exogenously hormones as well as endophytic extracts had varying effects on the hormonal content of the common bean plants, and some of these treatments caused a significant increase in the hormonal content of the plant. The maximum increase in the plant contents of IAA, GA3, and BA (0.8 ± 0.5, 18.4 ± 1.3, and 3.4 ± 0.1 mg/100 g fresh weight (FW), respectively) was achieved due to treatment by the metabolites of *B. thuringiensis* (PB2), while the foliar spray using metabolites of *A. sorghi* (PF2) led to the maximum increase in ABA (6.2 ± 0.5 µg/100 g) (Table 6).

In a comparative study, El-Mergawi and El-Wahed [92] conducted a greenhouse experiment to determine how foliar sprays of IAA or salicylic acid (SA) affect maize plant growth and the concentrations of endogenous IAA and SA. They reported that spraying plants with IAA and SA (0.25–2 mM) significantly increases the endogenous content of IAA and SA in a concentration-dependent manner, and the maximum increase (10–34%) was recorded after two days of treatment. Hormones applied exogenously alter plant growth and development by causing changes in their intrinsic contents. However, it is unclear whether exogenous hormones have direct effects on growth or are linked to changes in endogenous hormones [93].

Regarding endophytic extracts, the culture filtrates of the endophytic fungi *Fusarium proliferatum* BRL1 and *Asprgillus fumigatus* TS1 were examined for their plant growth-promoting traits on the mutant rice cultivar Waito-C. It was demonstrated that these culture filtrates improve biomass, chlorophyll content, and shoot-root length. In addition, a considerable increase in endogenous gibberellin (GA_1_) has been reported in treated rice plants [94]. Our results might be attributed to the master role of IAA in promoting plant development and growth by inducing various processes involving apical dominance, tissue growth, cell division, differentiation of vascular tissues, senescence and ripening, gravitropism and phototropism, embryogenesis, and lateral root initiation [82]. In addition, BA stimulates plant cell division, promotes plant growth, sets blossoms, and improves fruit quality [95].

The reason the IAA concentration was sometimes higher/lower or the same in the control and IAA-treated plants (Table 6) could be attributed to PGPR influencing plant auxin homeostasis by various mechanisms depending on the strains of bacteria. In addition, PGPR can affect the expression of the plant genes that are part of auxin synthesis, transport, or signaling machinery. For instance, PGPR *Pseudomonas putida* 1290 can utilize IAA as a nutritive substrate, thus eliminating the inhibiting effect of high levels of exogenous IAA on plant. The extracellular applications of both IAA and IAA-producing strains was used as a source of exogenous IAA [96].

Furthermore, PGPR can synthesize substances with auxin-like activity such as cyclopeptides and indole. Cyclopeptides stimulate the expression of auxin-induced marker constructs DR5/uidA and BA3/uidA and cause the degradation of Aux/IAA, the repressor of auxin-dependent genes, enhancing auxin signal transduction [97]. A small fraction of the indole that is produced by the PGPR can be converted into IAA. Interestingly, indole at higher concentrations can act as an auxin antagonist [98].

In addition, some biologically active substances produced by PGPR do not have auxin-like activity but can still affect plant auxin homeostasis such as the volatile compounds acetoin, 3,4-butanediol [99], albuterol and 1,3- propanediol, which can affect the expression of IAA transporter genes and can enhance the expression of auxin synthesis genes [100].

In a greenhouse, a soya bean seedling was inoculated with *Porostereum spadiceum* AGH786 endophytic fungus under NaCl stress. Phytohormones such as GAs, JA, and ABA, as well as isoflavones, tended to be secreted, but GAs was secreted in greater quantities than in the control plants [101]. Different phytohormones produced by endophytes reduce the dependence on synthetic fertilizers.

#### 3.3.6. Effect of the Foliar Spraying of Exogenously Applied Hormones and Microbial Endophyte Metabolites on the Yield Parameters of Common Bean Plants

In the current study, yield analysis demonstrated that all treatments (IAA, BA, PB2, PB5, BM, PF2, PF3, and FM) caused a significant increase in pod count, seed count, and protein content; the maximum increase in these parameters was recorded for the FM treatment. In addition, IAA and all endophytic metabolites significantly increased pods and seeds weight, with a maximum increase recorded for *Brevibacillus agri* PB5 treatment.

The exogenous application of IAA (100 ppm) caused significant increases in the weight of 100 seeds, while the outcomes of the other treatments seemed similar to that of the control. By contrast, all treatments significantly increased the carbohydrate content of the treated common beans, and the maximum increase was assigned for the *Brevibacillus agri* PB5 treatment (Table 7).

Another experiment was carried out to see how different growth regulators affected the growth and yield of lentil plants. Giannakoula and colleagues reported that plants treated with gibberellic acid showed a 26% reduction in the 1000-seed weight. Spraying plants with prohexadione-calcium and topflor, on the contrary, increased the weight of 1000 seeds by 16% percent and 30%, respectively [102]. It has been observed that the application of culture filtrate of the endophytic fungus *Piriformospora indica* to pots containing *Helianthus annus* Sun gold plants and growing plants for 90 days in a greenhouse increased the seed number, seed dry weight, and seed oil content by 9.12%, 45.89% and 51.13% respectively, in addition to increasing the total biomass (roots, stems, leaves, flowers, and seeds) by 36.7% compared to the control [103]. Several studies have reported that endophytes enhance legume crop yield by producing phytohormones such as IAA and GA3 [104], and ethylene [105].

## 4. Conclusions

Endophytic microbes have been shown to play an important role in plant growth, development, and fitness by producing growth-promoting substances and secondary metabolites. Therefore, six bacterial and four fungal endophytes were isolated from the sterilized roots of the *Phaseolus vulgaris* plant, and they were subjected to molecular identification based on the amplification and sequencing of bacterial 16S rRNA and the fungal ribosomal DNA internal transcribed spacer (ITS) region. The obtained microbial species exhibited plant growth-promoting activities, including phosphate solubilizing, ammonia production, nitrogen fixation, biocontrol of phytopathogen, extracellular enzymatic activities, and IAA production. A field trial was conducted to investigate the effect of two exogenously plant growth-promoting hormones (IAA and BA) compared to the metabolites of the most potent bacterial (*Bacillus thuringiensis* PB2 and *Brevibacillus agri* PB5) and fungal (*Alternaria sorghi* PF2, *Penicillium commune* PF3) endophytic species individually or in combination (BM and FM) as a mode of foliar spraying on the growth and biochemical properties of common bean plants in order to evaluate the possibility of applying these microbial isolates as enhancers for legume plants. This study represents the first investigation of the efficacy of microbial endophytes as plant growth-promoters in comparison to exogenously applied hormones. Our investigations demonstrated that bacterial and fungal endophytic metabolites surpassed the exogenously applied hormones in increasing the plant biomass, photosynthetic pigments, carbohydrate and protein contents, antioxidant enzyme activity, endogenous hormones, and yield traits. Our findings illustrate that the endophyte *Brevibacillus agri* (PB5) had the greatest effect on both the vegetative growth and metabolism of the common bean plants. In the future, the use of endophytic microbes for plant growth promotion will eliminate the need for organic fertilizers and synthetic growth regulators in various crops. More research on different plant species is required to see whether these are plant-specific characteristics and to learn more about the processes at work in the interactions between microbial metabolites and the host that enable plants to optimize their responses under aggressive conditions.

## Figures and Tables

**Table 1:** The 16S rRNA and ITS sequence identifications of the bacterial and fungal endophytes isolated from sterilized root of common bean plants.

Microbial Strain Code	GenBank Accession Number	Homolog Sequences	Sequence Identity (%)	Closest Accession Number
PB1	MW485939	*Enterobacter asburiae*	99.16	BBED01000197
PB2	MW485940	*Bacillus thuringiensis*	99.69	ACNF01000156
PB3	MW485941	*Acinetobacter radioresistens*	99.70	BAGY01000082
PB4	MW485942	*Brevibacillus brevis*	98.88	AB271756
PB5	MW485943	*Brevibacillus agri*	99.84	D78454
PB6	MW485944	*Bacillus subtilis*	98.99	ABQL01000001
PF1	MW485935	*Penicillium crustosum*	99.62	NR077153
PF2	MW485936	*Alternaria sorghi*	98.63	NR160246
PF3	MW485937	*Penicillium commune*	98.69	NR111143
PF4	MW485938	*Aspergillus flavus*	98.84	NR111041

**Table 2:** The antagonistic activity of isolated endophytes against selected phytopathogenic fungi.

Endophytic Isolates	Percentage of Growth Inhibition (%)			
	*F. oxysporum*	*A. alternata*	*V. dahlia*	*P. ultimum*
Control	0 ^i^	0 ^h^	0 ^h^	0 ^h^
PB1	31.6 ± 1.4 ^g^	45.6 ± 0.8 ^c^	28.6 ± 0.3 ^f^	12.3 ± 0.8 ^f^
PB2	54 ± 0.57 ^c^	49 ± 1.1 ^b^	57.6 ± 2 ^b^	24.3 ± 1.4 ^d^
PB3	24.6 ± 0.33 ^h^	34.6 ± 0.8 ^f^	22.6 ± 1.4 ^g^	13.3 ± 1.4 ^ef^
PB4	41.6 ± 1.4 ^e^	44.6 ± 0.8 ^c^	33.6 ± 0.8 ^e^	15 ± 1.1 ^e^
PB5	58.6 ± 0.6 ^ab^	51.6 ± 0.5 ^a^	62.6 ± 2 ^a^	22.6 ± 2.6 ^d^
PB6	26.6 ± 2 ^h^	21 ± 0.57 ^g^	36.3 ± 0.3 ^d^	9.6 ± 0.3 ^g^
FP1	46.6 ± 1.45 ^d^	37.3 ± 1.2 ^e^	21.6 ± 1.3 ^g^	41 ± 1.7 ^c^
FB2	57.3 ± 0.6 ^b^	42.3 ± 0.8 ^d^	38.6 ± 0.8 ^c^	55.3 ± 2.6 ^a^
FB3	61.3 ± 2.3 ^a^	45.6 ± 1.4 ^c^	31.6 ± 1.4 ^e^	48.3 ± 3.1 ^b^
FB4	35.6 ± 1.4 ^f^	33.3 ± 0.8 ^f^	26.6 ± 0.8 ^f^	24.6 ± 0.8 ^d^

Values in the same column with different letters are significantly different (*p* ≤ 0.05), values refer to the means ± SE (*n* = 3).

**Table 3:** Effect of foliar spraying of exogenously applied hormone and microbial endophyte metabolites on root, shoot length and leave numbers of common bean plants.

Treatments		Shoot Length (cm)		Root Length (cm)		Leaf Numbers	
		1st Stage	2nd Stage	1st Stage	2nd Stage	1st Stage	2nd Stage
Control		22.4 ± 0.5 ^b^	27.4 ± 0.3 ^ab^	9.3 ± 0.6 ^ab^	8.98 ± 0.3 ^b^	5.0 ± 0.0 ^a^	5.67 ± 0.2 ^a^
Exogenously Applied Hormones	IAA	23.3 ± 0.5 ^b^	29.3 ± 0.9 ^ab^	8.0 ± 0.4 ^b^	9.8 ± 0.7 ^ab^	4.6 ± 0.3 ^a^	6.0 ± 0.0 ^a^
	BA	25.3 ± 0.4 ^a^	28.2 ± 0.7 ^ab^	9.7 ± 0.4 ^ab^	8.9 ± 0.4 ^b^	4.4 ± 0.2 ^a^	6.0 ± 0.0 ^a^
Bacterial Endophytes	PB2	26.9 ± 0.8 ^a^	34.0 ± 0.9 ^a^	10.03 ± 0.3 ^ab^	11.5 ± 0.6 ^a^	4.6 ± 0.2 ^a^	6.0 ± 0.0 ^a^
	PB5	28.7 ± 0.9 ^a^	33.8 ± 1.6 ^a^	9.4 ± 0.4 ^ab^	10.5 ± 0.9 ^ab^	4.6 ± 0.3 ^a^	6.0 ± 0.0 ^a^
	BM	25.1 ± 0.8 ^a^	25.5 ± 0.7 ^b^	9.5 ± 0.2 ^ab^	11.5 ± 0.4 ^a^	4.1 ± 0.1 ^a^	6.0 ± 0.0 ^a^
Fungal Endophytes	PF2	26.1 ± 0.4 ^a^	29.2 ± 0.7 ^ab^	8.4 ± 0.3 ^b^	12.7 ± 0.7 ^a^	4.0 ± 0.0 ^a^	6.0 ± 0.0 ^a^
	PF3	26.1 ± 0.7 ^a^	25.5 ± 0.3 ^b^	10.2 ± 0.5 ^ab^	12.5 ± 0.5 ^a^	4.1 ± 0.1 ^a^	6.0 ± 0.0 ^a^
	FM	25.3 ± 0.7 ^a^	27.2 ± 0.8 ^ab^	10.4 ± 0.3 ^ab^	11.0 ± 0.5 ^a^	4.0 ± 0.0 ^a^	6.0 ± 0.0 ^a^

Different letters between columns denote that mean values are significantly different (*p* ≤ 0.05) by Tukey’s test.

**Table 4:** Effect foliar spraying of exogenously applied hormones and microbial endophyte metabolites on carbohydrates and protein contents of common bean plants.

Treatments		Root Carbohydrate (mg g^−^^1^ DW)		Root Protein (mg g^−^^1^ DW)		Shoot Carbohydrate (mg g^−^^1^ DW)		Shoot Protein (mg g^−^^1^ DW)	
		1st Stage	2nd Stage	1st Stage	2nd Stage	1st Stage	2nd Stage	1st Stage	2nd Stage
Control		90.7 ± 0.4 ^c^	70.4 ± 0.7 ^e^	58.8 ± 0.6 ^b^	60.9 ± 0.9 ^b^	75.7 ± 1.2 ^d^	77.2 ± 1.5 ^d^	49.8 ± 0.3 ^d^	56.4 ± 0.7 ^b^
Exogenously Applied Hormones	IAA	108.4 ± 1.7 ^b^	86.6 ± 1.1 ^d^	59.8 ± 0.7 ^b^	64.2 ± 0.9 ^b^	98.7 ± 0.6 ^c^	101.9 ± 2.9 ^c^	53.7 ± 0.4 ^c^	59.3 ± 0.8 ^a^
	BA	113.2 ± 3.2 ^ab^	90.6 ± 4.2 ^cd^	61.2 ± 0.8 ^b^	67.4 ± 1.5 ^ab^	115.6 ± 3.5 ^ab^	112.4 ± 1.4 ^ab^	65.5 ± 0.3 ^a^	59.3 ± 0.4 ^a^
Bacterial Endophytes	PB2	108.4 ± 1.7 ^bb^	99.5 ± 0.7 ^bc^	61.1 ± 0.7 ^b^	64.6 ± 0.9 ^b^	113.2 ± 4.5 ^ab^	107.6 ± 4.9 ^bc^	58.6 ± 0.7 ^b^	60.7 ± 1.1 ^a^
	PB5	118.2 ± 1.7 ^a^	95.5 ± 1.4 ^c^	64.8 ± 0.1 ^a^	69.3 ± 1.3 ^a^	110.0 ± 5.5 ^b^	117.3 ± 2.4 ^ab^	58.9 ± 0.6 ^b^	59.8 ± 0.2 ^a^
	BM	107.6 ± 3.7 ^b^	105.9 ± 3.5 ^b^	64.9 ± 0.6 ^a^	70.8 ± 0.9 ^a^	122.9 ± 2.02 ^a^	116.5 ± 1.6 ^ab^	59.0 ± 0.5 ^b^	59.3 ± 0.6 ^a^
Fungal Endophytes	PF2	103.5 ± 4.5 ^b^	121.4 ± 4.2 ^a^	61.5 ± 0.8 ^b^	66.2 ± 0.8 ^b^	119.8 ± 6.9 ^a^	111.2 ± 1.2 ^ab^	58.3 ± 0.9 ^b^	59.8 ± 0.3 ^a^
	PF3	104.4 ± 2.1 ^b^	106.1 ± 2.9 ^b^	64.01 ± 0.8 ^a^	69.85 ± 1.21 ^a^	103.6 ± 2.9 ^b^	107.6 ± 4.9 ^bc^	59.3 ± 0.4 ^b^	60.3 ± 0.2 ^a^
	FM	109.9 ± 2.8 ^b^	110.03 ± 2.8 ^ab^	63.9 ± 0.8 ^a^	70.7 ± 1.5 ^a^	113.2 ± 4.5 ^ab^	120.5 ± 0.7 ^a^	58.9 ± 0.6 ^b^	60.3 ± 0.4 ^a^

Different letters between columns denote that mean values are significantly different (*p* ≤ 0.05) by Tukey’s test.

**Table 5:** Effect of foliar spraying of exogenously applied hormones and microbial endophyte metabolites on amylase, protease, catalase, and peroxidase of common bean plants.

Treatments		Amylase (Unit/µg FW)		Protease (Unit/µg FW)		Catalase (Unit/µg FW)		Peroxidase (Unit/µg FW)	
		1st Stage	2nd Stage	1st Stage	2nd Stage	1st Stage	2nd Stage	1st Stage	2nd Stage
Control		1.2 ± 0.1 ^b^	1.3 ± 0.2 ^c^	0.14 ± 0.01 ^a^	0.15 ± 0.0 ^b^	66.7 ± 1.4 ^a^	95.0 ± 2.9 ^c^	58.33 ± 1.7 ^d^	58.33 ± 1.7 ^c^
Exogenously Applied Hormones	IAA	1.7 ± 0.1 ^b^	1.9 ± 0.02 ^b^	0.15 ± 0.01 ^a^	0.18 ± 0.0 ^ab^	82.0 ± 1.7 ^b^	106.6 ± 1.7 ^b^	95.00 ± 5.0 ^ab^	96.67 ± 3.3 ^b^
	BA	1.8 ± 0.1 ^b^	1.9 ± 0.1 ^b^	0.14 ± 0.0 ^a^	0.16 ± 0.01 ^b^	63.3 ± 1.3 ^a^	63.3 ± 3.3 ^d^	101.6 ± 1.7 ^a^	105.0 ± 2.9 ^a^
Bacterial Endophytes	PB2	2.7 ± 0.2 ^a^	2.9 ± 0.1 ^a^	0.16 ± 0.01 ^a^	0.15 ± 0.01 ^b^	83.3 ± 0.4 ^b^	123.3 ± 3.3 ^b^	86.7 ± 3.3 ^b^	103.3 ± 1.7 ^a^
	PB5	2.7 ± 0.1 ^a^	3.1 ± 0.1 ^a^	0.17 ± 0.0 ^a^	0.19 ± 0.0 ^a^	88.3 ± 1.7 ^b^	155.0 ± 5.0 ^a^	101.7 ± 1.7 ^a^	111.6 ± 4.4 ^a^
	BM	1.7 ± 0.1 ^b^	2.8 ± 0.3 ^a^	0.15 ± 0.0 ^a^	0.19 ± 0.0 ^a^	83.3 ± 1.4 ^b^	113.3 ± 3.3 ^b^	103.3 ± 1.7 ^a^	96.67 ± 3.3 ^b^
Fungal Endophytes	PF2	2.13 ± 0.2 ^a^	1.9 ± 0.1 ^b^	0.18 ± 0.0 ^a^	0.18 ± 0.01 ^ab^	86.7 ± 0.3 ^b^	111.6 ± 4.4 ^b^	76.67 ± 1.7 ^c^	103.3 ± 3.3 ^ab^
	PF3	1.7 ± 0.1 ^b^	2.2 ± 0.1 ^b^	0.18 ± 0.0 ^a^	0.18 ± 0.01 ^ab^	75.0 ± 0.4 ^c^	83.33 ± 4.4 ^c^	98.33 ± 4.4 ^a^	91.67 ± 1.7 ^b^
	FM	1.76 ± 0.1 ^b^	2.3 ± 0.1 ^b^	0.15 ± 0.0 ^a^	0.18 ± 0.0 ^ab^	85.0 ± 0.9 ^b^	91.67 ± 1.7 ^c^	38.33 ± 4.4 ^e^	66.67 ± 4.4 ^c^

Different letters between columns denote that mean values are significantly different (*p* ≤ 0.05) by Tukey’s test.

**Table 6:** Effect of exogenous application of exogenously applied hormones and microbial endophyte metabolites on endogenous levels of indole-3-acetic acid (IAA), benzyl Adenine (BA), gibberellic acid (GA3), and abscisic acid (ABA) of common bean plants.

Treatments		IAA (mg/100 g FW)	BA (mg/100 g FW)	GA3 (mg/100 g FW)	ABA (µg/100 g FW)
Control		0.3 ± 0.02 ^b^	1.3 ± 0.7 ^cd^	2.6 ± 0.9 ^c^	1.02 ± 0.7 ^c^
Exogenously Applied Hormones	IAA	0.3 ± 0.05 ^b^	0.85 ± 0.6 ^d^	14.8 ± 2.4 ^b^	2.1 ± 0.3 ^c^
	BA	0.4 ± 0.05 ^ab^	1.9 ± 0.7 ^bc^	3.2 ± 1.7 ^c^	1.6 ± 0.6 ^c^
Bacterial Endophytes	PB2	0.8 ± 0.5 ^a^	3.4 ± 0.1 ^a^	18.4 ± 1.3 ^a^	2.2 ± 0.5 ^c^
	PB5	0.4 ± 0.1 ^b^	2.0 ± 0.8 ^b^	8.3 ± 1.9 ^d^	2.7 ± 0.2 ^c^
	BM	0.31 ± 0.06 ^b^	1.4 ± 0.5 ^c^	3.8 ± 1.9 ^c^	4.1 ± 0.3 ^b^
Fungal Endophytes	PF2	0.4 ± 0.04 ^b^	2.6 ± 0.6 ^ab^	16.9 ± 1.02 ^ab^	6.2 ± 0.5 ^a^
	PF3	0.34 ± 0.04 ^b^	1.8 ± 0.1 ^bc^	17.1 ± 1.7 ^a^	5.7 ± 0.7 ^ab^
	FM	0.6 ± 0.05 ^a^	3.0 ± 0.9 ^a^	2.9 ± 1.5 ^c^	4.3 ± 0.8 ^b^

Different letters between columns denote that mean values are significantly different (*p* ≤ 0.05) by Tukey’s test.

**Table 7:** Effect of foliar spraying of exogenously applied hormones and microbial endophyte metabolites on yield parameters and seed yield components of common bean plants.

Treatments		Pods/Plant		Seeds/Pod		100 Seeds Weight	Seed Yield Components	
		Number	Weight (g)	Number	Weight (g)	Weight (g)	Carbohydrate (mg/g DW)	Protein (mg/g DW)
Control		2.0 ± 0.02 ^c^	7.3 ± 0.1 ^b^	6.3 ± 0.3 ^d^	6.0 ± 0.0 ^b^	45.2 ± 4.8 ^b^	81.5 ± 0.9 ^c^	54.6 ± 0.2 ^b^
Exogenously applied Hormones	IAA	3.0 ± 0.6 ^b^	8.8 ± 0.4 ^ab^	11.3 ± 2.03 ^c^	7.7 ± 0.3 ^ab^	71.2 ± 9.9 ^a^	110.8 ± 3.5 ^b^	60.1 ± 1.0 ^a^
	BA	3.7 ± 0.3 ^ab^	7.7 ± 0.3 ^b^	13.7 ± 0.9 ^b^	6.7 ± 0.3 ^b^	49.3 ± 4.7 ^b^	123.1 ± 4.3 ^ab^	63.5 ± 1.9 ^a^
Bacterial Endophytes	PB2	4.0 ± 0.1 ^a^	8.5 ± 0.3 ^ab^	15.0 ± 0.6 ^b^	7.1 ± 0.1 ^ab^	47.6 ± 1.2 ^b^	134.6 ± 2.1 ^a^	64.9 ± 1.2 ^a^
	PB5	4.7 ± 0.3 ^a^	10.4 ± 0.2 ^a^	18.0 ± 0.6 ^a^	9.0 ± 0.1 ^a^	50.1 ± 1.3 ^b^	138.9 ± 1.9 ^a^	62.8 ± 0.1 ^a^
	BM	4.7 ± 0.3 ^a^	8.7 ± 0.3 ^ab^	16.3 ± 2.2 ^ab^	7.7 ± 0.3 ^ab^	48.3 ± 5.1 ^b^	120.7 ± 0.7 ^b^	64.9 ± 1.2 ^a^
Fungal Endophytes	PF2	5.0 ± 1.0 ^a^	8.9 ± 0.2 ^ab^	18.7 ± 0.9 ^a^	7.7 ± 0.3 ^ab^	41.3 ± 3.02 ^b^	118.4 ± 1.2 ^b^	61.2 ± 0.8 ^a^
	PF3	5.3 ± 0.3 ^a^	9.5 ± 0.4 ^a^	17.0 ± 2.7 ^ab^	8.3 ± 0.3 ^a^	51.6 ± 8.3 ^b^	127.5 ± 4.5 ^ab^	65.9 ± 0.5 ^a^
	FM	5.4 ± 0.3 ^a^	9.9 ± 0.3 ^a^	20.3 ± 0.3 ^a^	8.7 ± 0.3 ^a^	42.7 ± 2.3 ^b^	107.6 ± 3.7 ^b^	67.1 ± 1.3 ^a^

Different letters between columns denote that mean values are significantly different (*p* ≤ 0.05) by Tukey’s test.

## Data Availability

The data presented in this study are available on request from the corresponding author.

## Supplementary Materials

The following are available online at https://www.mdpi.com/article/10.3390/cells10051059/s1, Table S1: Extracellular enzymatic activities of bacterial and fungal endophytic strains isolated from roots of common bean plants. Table S2: Effect of foliar spraying of exogenously applied hormones and microbial endophyte metabolites on fresh and dry weight of shoot and root of common bean plants. Table S3: Effect of foliar spraying of exogenously applied hormones and microbial endophyte metabolites on chlorophyll and carotene content of common bean plants. Figure S1. Example of plant growth-promoting activities of some endophytic bacteria and fungi. (A) ammonia production; (B) phosphate solubilization; (C and D) amylase activity for some bacterial and fungal strains; (E) Protease activity for some bacterial strain; (F) cellulase activity for some fungal strains; (G) Xylanase activity for some bacterial strains. PB1 is *E. asburiae;* PB2 is *B. thuringiensis*; PB3 is *A. radioresistens*; PB4 is *B. brevis*; PB5 is *B. agri*; PB6 is *B. subtilis*; PF1 is *P. crustosum*; PF2 is *A. sorghi*; PF3 is *P. commune*; PF4 is *A. flavus.* Figure S2: In vitro antagonistic activity of some endophytic bacterial species obtained in the current study against against *Fusarium oxysporum* (A), *Pythium ultimum* (B), and *Alternaria alternata* (C) as plant pathogenic fungi. The selected endophytic bacterial species denotes as (A) *Brevibacillus agri PB5;* (B) *Bacillus thuringiensis* PB2; (C) *Brevibacillus brevis* PB4; (D) *Brevibacillus agri PB5;* (E) *Acinetobacter radioresistens* PB3; (F) *Brevibacillus agri PB5*. Figure S3. (A) photo of greenhouse experiemnt; (B) photo showed gradually shhot and root length for different treatment.
