# Supplementary material for: Comparative Study between Exogenously Applied Plant Growth Hormones versus Metabolites of Microbial Endophytes as Plant Growth-Promoting for Phaseolus vulgaris L
Source: Cells. 2021 Apr 29;10(5):1059. doi: 10.3390/cells10051059 (PMC8146795; doi:10.3390/cells10051059)
Supplement: Supplementary file 1 [file cells-10-01059-s001.zip › cells-1168379-SI.pdf]

cells

Article (Supplementary Data)

Comparative Study between Exogenously Applied Plant Growth Hormones versus Metabolites of Microbial Endophytes as Plant Growth-Promoting for *Phaseolus vulgaris* L.

Mohamed A. Ismail [1], Mohamed A. Amin [1], Ahmed M. Eid [1], Saad El-Din Hassan [1,*], Hany A. M. Mahgoub [1], Islam Lashin [1,2], Abdelrhman T. Abdelwahab [1,3], Ehab Azab [4], Adil A. Gobouri [5], Amr Elkelish [6,7] and Amr Fouda [1,*]

Supplementary materials:

Table S1: Extracellular enzymatic activities of bacterial and fungal endophytic strains isolated from roots of common bean plants.

Endophytic isolates	Ammonia production	P solubilization Diameter of a clear zone (mm)	Enzyme production (diameter of clear zones (mm)				
			Amylase	Cellulase	Protease	Pectinase	Xylanase
PB1	-	0^{g}	17.6 ± 0.3^{d}	13.3 ± 0.3^{g}	17.3 ± 0.3^{g}	0^{i}	12.6 ± 0.3^{g}
PB2	+	7.6 ± 0.23^{d}	17.6 ± 0.3^{d}	21.3 ± 0.6^{d}	26 ± 0.57^{e}	12.6 ± 0.8^{h}	14.3 ± 0.3^{f}
PB3	-	0^{g}	13.6 ± 0.3^{f}	15.6 ± 0.8^{f}	0^{h}	24 ± 1.5^{e}	21.3 ± 0.8^{d}
PB4	++	0^{g}	0^{h}	18.3 ± 0.8^{e}	24 ± 0.57^{f}	14.3 ± 0.3^{g}	8.6 ± 0.8^{h}
PB5	++	8.9 ± 0.26^{c}	15 ± 0.57^{e}	21.3 ± 0.8^{d}	28.6 ± 0.88^{d}	11.6 ± 0.6^{h}	15.3 ± 0.3^{e}
PB6	-	5.2 ± 0.17^{f}	12 ± 0.57^{g}	18.3 ± 0.3^{e}	0^{h}	15.6 ± 0.6^{f}	14 ± 0.6^{f}
FP1	++	0^{g}	33 ± 1.15^{b}	40 ± 1.15^{a}	22.6 ± 0.88^{f}	35.3 ± 1.2^{c}	24 ± 1^{c}
FB2	++	11 ± 0.32^{a}	37 ± 1.7^{a}	42 ± 1^{a}	38.3 ± 1.2^{a}	42.3 ± 1.3^{a}	31.3 ± 0.8^{a}
FB3	++	9.9 ± 0.43^{b}	30 ± 1.1^{c}	36.6 ± 1.7^{b}	34.3 ± 1.7^{b}	38.6 ± 1.4^{b}	27.3 ± 0.8^{b}
FB4	-	6.5 ± 0.28^{e}	28.3 ± 1.2^{c}	27.3 ± 1.4^{c}	30.6 ± 1.2^{c}	33 ± 1^{d}	0^{i}

Values within the same column with different letters are significantly different ($p \leq 0.05$), values are means ± SE (n = 3). + and ++ meaning the low and high ammonia production respectively based on color change. PB1 is *E. asburiae;* PB2 is *B. thuringiensis*; PB3 is *A. radioresistens*; PB4 is *B. brevis*; PB5 is *B. agri*; PB6 is *B. subtilis*; PF1 is *P. crustosum*; PF2 is *A. sorghi*; PF3 is *P. commune*; PF4 is *A. flavus*.

Table S2: Effect of foliar spraying of exogenously applied hormones and microbial endophyte metabolites on the fresh and dry weight of shoot and root of common bean plants.

Treatments		Shoot FW (g)		Shoot DW (g)		Root FW (g)		Root DW (g)	
		1st stage	2nd stage	1st stage	2nd stage	1st stage	2nd stage	1st stage	2nd stage
Control		2.4±0.1^{b}	5.04±0.1^{c}	0.27±0.0^{d}	0.6±0.06^{c}	0.4±0.02^{b}	0.5±0.**02**^{b}	0.05±0.002^{b}	0.08±0.002^{b}
Exogenously Applied Hormones	IAA	2.5±0.2^{cb}	7.7±0.9^{b}	0.4±0.0^{b}	0.8±0.04^{c}	0.5±0.005^{a}	**0.61**±0.02^{a}	0.07±0.001^{a}	0.09±0.001^{b}
	BA	3.3±0.2^{ba}	7.6±0.9^{b}	0.3±0.01^{c}	1.01±0.1^{b}	0.52±0.007^{a}	**.07**±0.05^{a}	0.08±0.002^{a}	0.09±0.005^{ab}
Bacterial Endophytes	PB2	3.3±0.2^{ba}	12.5±1.02^{a}	0.5±0.01^{b}	1.4±0.02^{a}	0.5±0.01^{ab}	**.07**±0.1^{a}	0.08±0.004^{a}	0.1±0.01^{a}
	PB5	4.9±0.3^{a}	11.8±0.7^{a}	0.6±0.02^{a}	1.3±0.01^{a}	0.59±0.02^{ab}	**.08**±0.04^{a}	0.07±0.001^{a}	0.13±0.01^{a}
	BM	3.8±0.4^{a}	9.3±0.7^{ab}	0.5±0.01^{b}	1.4±0.03^{a}	0.5±0.012^{ab}	0.7±0.05^{a}	0.05±0.001^{b}	0.14±0.005^{a}
Fungal Endophytes	PF2	3.8±0.3^{a}	12.2±0.8^{a}	0.5±0.02^{b}	1.5±0.05^{a}	0.5±0.006^{ab}	**0.74±0.05**^{a}	0.04±0.001^{b}	0.1±0.001^{a}
	PF3	3.9±0.2^{a}	10.3±0.4^{ab}	0.5±0.01^{b}	1.1±0.05^{b}	0.5±0.01^{ab}	**0.5**±0.01^{b}	0.07±0.002^{ab}	0.1±0.001^{ab}
	FM	3.5±0.2^{a}	10.7±0.3^{ab}	0.5±0.01^{b}	1.1±0.05^{b}	0.5±0.02^{ab}	**0.5**±0.**01**^{b}	0.1±0.001^{a}	0.09±0.002^{ab}

Different letters between columns denote that mean values are significantly different ($p \leq 0.05$) by Tukey's test. IAA is Indole-3-acetic acid; BA is Benzyl adenin; PB2 is *B. thuringiensis*; PB5 is *B. agri*; BM is bacterial mix; PF2 is *A. sorghi*; PF3 is *P. commune*; FM is fungal mix.

Table S3: Effect of foliar spraying of exogenously applied hormones and microbial endophyte metabolites on chlorophyll and carotene content of common bean plants.

Treatments		Chlorophyll a (mg/g FW)		Chlorophyll b (mg/g FW)		Total Chlorophyll (mg/g FW)		Total Carotene (mg/g FW)	
		1st stage	2nd stage	1st stage	2nd stage	1st stage	2nd stage	1st stage	2nd stage
Control		6.86±0.17^{b}	5.56±0.13^{b}	7.78±0.17^{ab}	16.25±0.17^{b}	14.6±0.14^{b}	21.81±0.10^{b}	1.79±0.25^{a}	0.92±0.09^{a}
**Exogenously Applied Hormones**	IAA	6.77±0.03^{b}	5.65±0.04^{b}	8.10±0.32^{ab}	20.28±0.19^{a}	14.8±0.35^{b}	25.92±0.15^{ab}	1.73±0.12^{a}	0.62±0.07^{b}
	BA	7.69±0.05^{a}	6.55±0.50^{a}	7.46±0.16^{b}	17.21±1.76^{ab}	15.1±0.20^{ab}	23.76±1.26^{b}	1.77±0.19^{a}	1.31±0.15^{a}
Bacterial Endophytes	PB2	6.78±0.25^{b}	5.41±0.13^{b}	9.36±0.61^{ab}	19.05±0.60^{ab}	16.1±0.37^{a}	24.46±0.62^{ab}	1.14±0.18^{a}	1.11±0.15^{a}
	PB5	6.78±0.1^{b}	5.86±0.14^{ab}	9.04±0.49^{ab}	20.50±0.40^{a}	15.8±0.38^{ab}	26.36±0.30^{a}	1.29±0.35^{a}	0.64±0.08^{b}
	BM	6.79±0.1^{b}	5.96±0.11^{ab}	8.82±0.28^{ab}	20.30±0.03^{a}	15.6±0.21^{ab}	26.26±0.11^{a}	1.48±0.16^{a}	0.77±0.02^{b}
Fungal Endophytes	PF2	6.79±0.1^{b}	5.90±0.14^{ab}	8.82±0.28^{ab}	18.52±2.08^{ab}	15.6±0.21^{ab}	24.41±1.95^{ab}	1.48±0.16^{a}	1.60±0.07^{a}
	PF3	6.87±0.05^{b}	6.12±0.08^{a}	9.54±0.37^{a}	20.00±0.93^{a}	16.4±0.34^{a}	26.12±0.86^{a}	1.34±0.22^{a}	1.53±0.03^{a}
	FM	6.92±0.01^{b}	6.20±0.03^{a}	8.98±0.16^{ab}	19.97±0.90^{ab}	15.9±0.18^{ab}	26.17±0.89^{a}	1.45±0.06^{a}	1.45±0.18^{a}

Values within the same column with different letters are significantly different ($p \leq 0.05$), values are means ± SE ($n = 3$). IAA is Indole-3-acetic acid; BA is Benzyl adenin; PB2 is *B. thuringiensis*; PB5 is *B. agri*; BM is bacterial mix; PF2 is *A. sorghi*; PF3 is *P. commune*; FM is fungal mix.

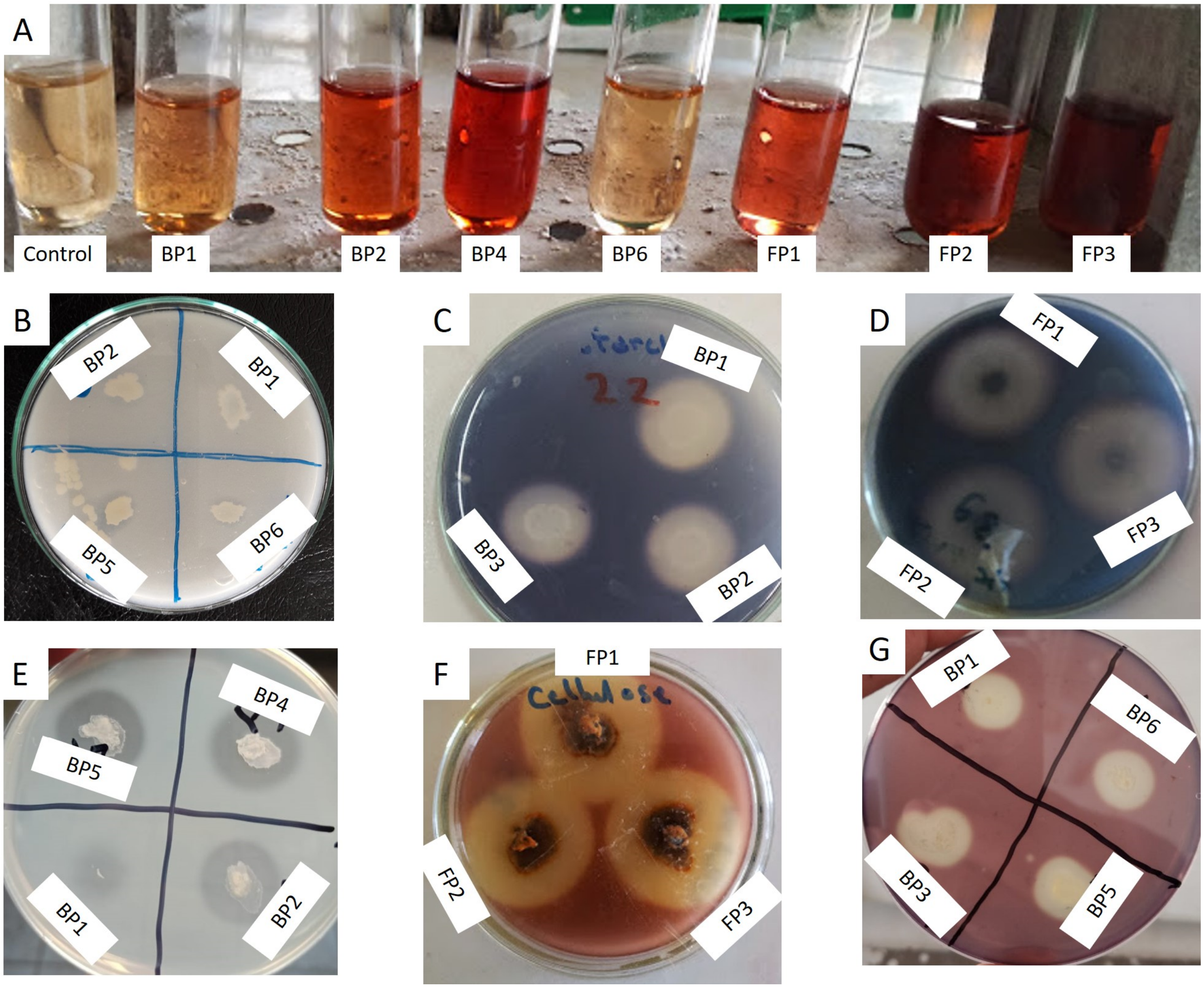

Figure S1. Example of plant growth-promoting activities of some endophytic bacteria and fungi. (A) ammonia production; (B) phosphate solubilization; (C and D) amylase activity for some bacterial and fungal strains; (E) Protease activity for some bacterial strain; (F) cellulase activity for some fungal strains; (G) Xylanase activity for some bacterial strains. PB1 is *E. asburiae;* PB2 is *B. thuringiensis*; PB3 is *A. radioresistens*; PB4 is *B. brevis*; PB5 is *B. agri*; PB6 is *B. subtilis*; PF1 is *P. crustosum*; PF2 is *A. sorghi*; PF3 is *P. commune*; PF4 is *A. flavus.*

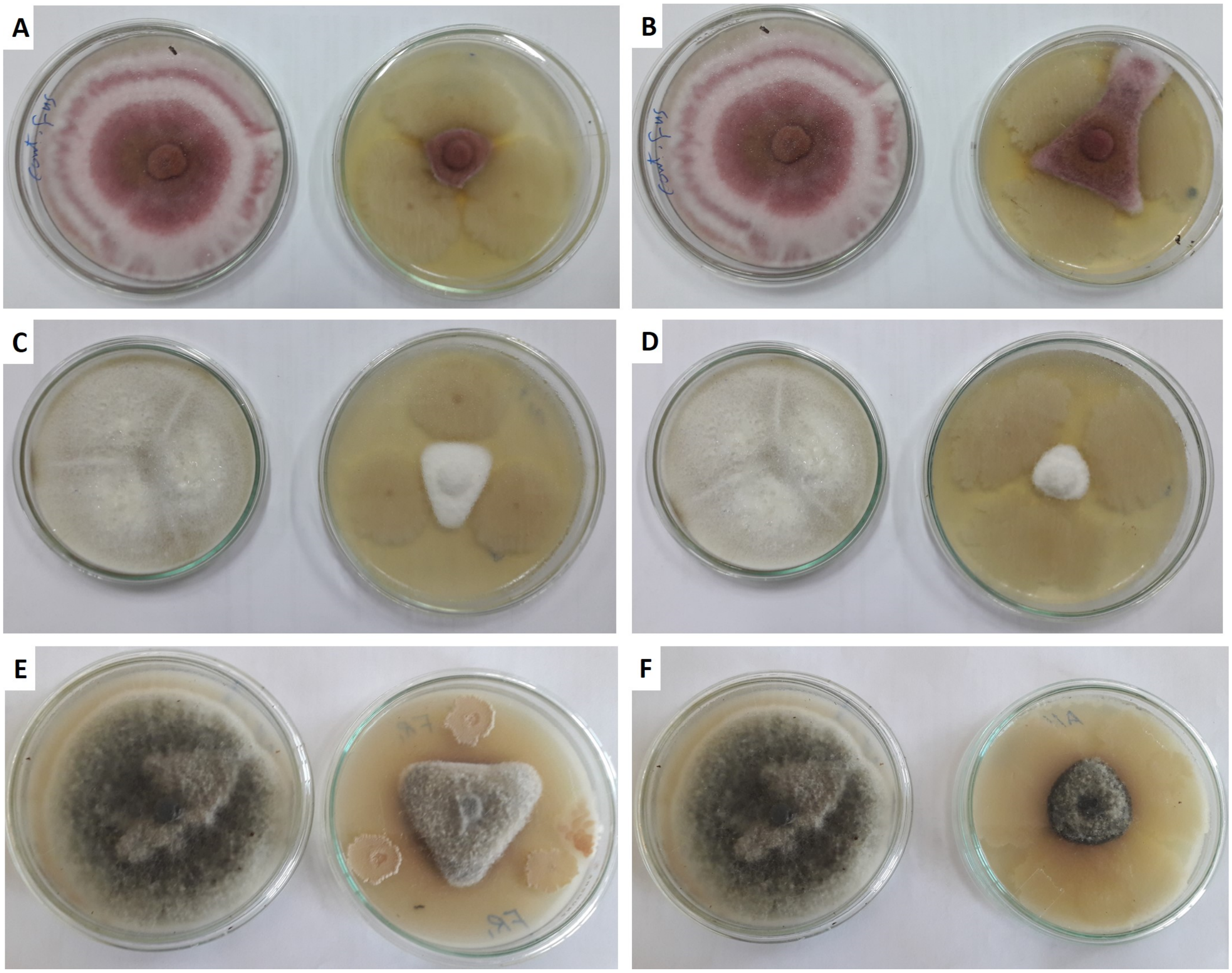

Figure S2. In vitro antagonistic activity of some endophytic bacterial species obtained in the current study against *Fusarium oxysporum* (A), *Pythium ultimum* (B), and *Alternaria alternata* (C) as plant pathogenic fungi. The selected endophytic bacterial species denotes as (A) *Brevibacillus agri PB5;* (B) *Bacillus thuringiensis* PB2; (C) *Brevibacillus brevis* PB4; (D) *Brevibacillus agri PB5;* (E) *Acinetobacter radioresistens* PB3; (F) *Brevibacillus agri PB5.*

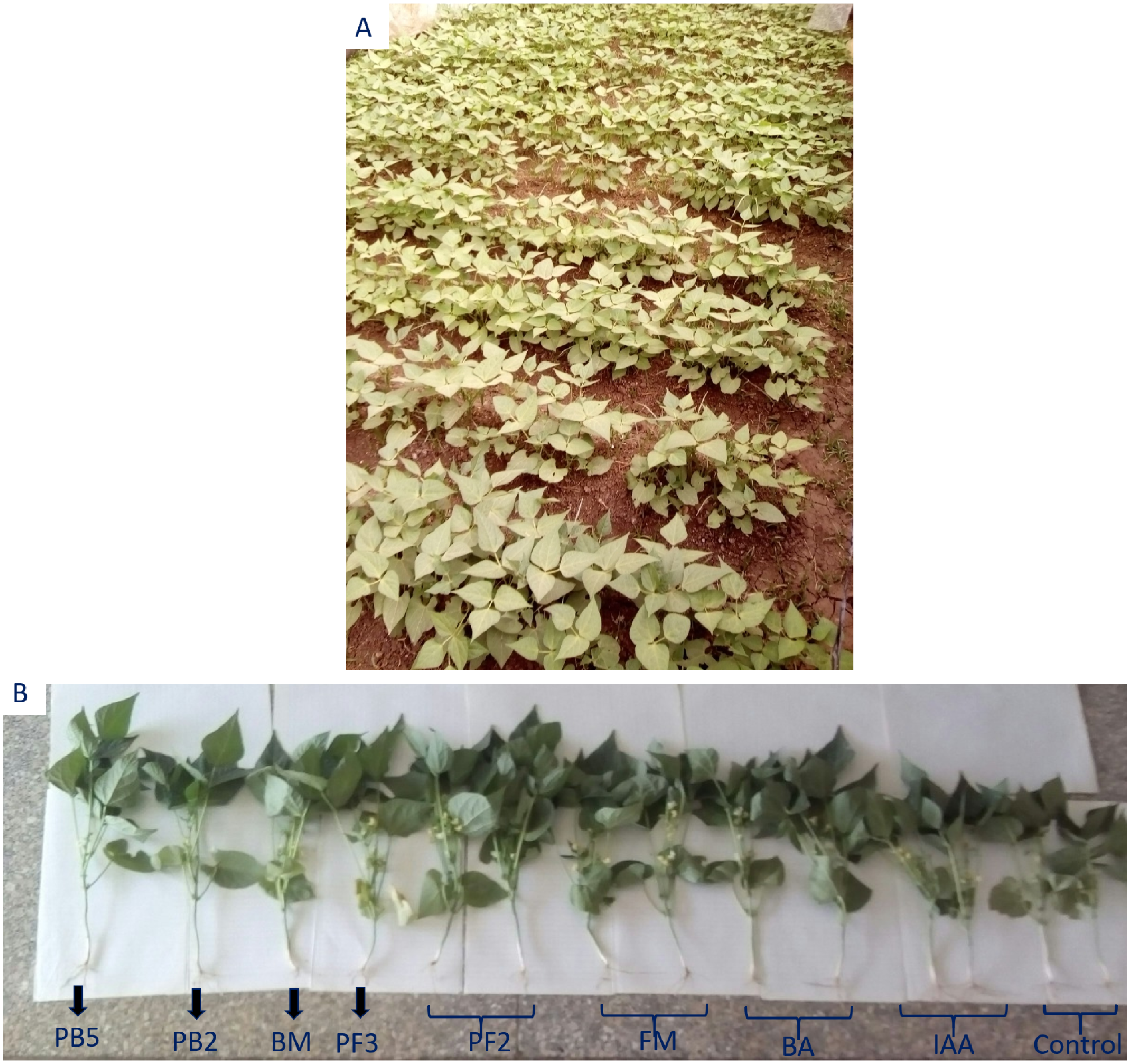

Figure S3. (A) photo of greenhouse experiemnt; (B) photo showed gradually shoot and root length for different treatment. IAA is Indole-3-acetic acid; BA is Benzyl adenin; PB2 is *B. thuringiensis*; PB5 is *B. agri*; BM is bacterial mix; PF2 is *A. sorghi*; PF3 is *P. commune*; FM is fungal mix.
